# Disruption of NF‐κB‐Mediated Copper Homeostasis Sensitizes Breast Cancer to Cuproptosis

**DOI:** 10.1002/advs.202506201

**Published:** 2025-09-26

**Authors:** Xiaomei Zhang, Yaqing Su, Weixiong Yang, Zimin Song, Zicheng Sun, Xueji Wu, Jianwen Chen, Bing Gao, Zekang Wang, Lei Wang, Qiwei Jiang, Lang Bu, Jingting Li, Ying Lin, Wei Xie, Jie Li, Jianping Guo

**Affiliations:** ^1^ Department of Breast and Thyroid Surgery the First Affiliated Hospital Sun Yat‐sen University Guangzhou Guangdong 510275 China; ^2^ Institute of Precision Medicine the First Affiliated Hospital Sun Yat‐sen University Guangzhou Guangdong 510275 China; ^3^ Department of Thoracic Surgery the First Affiliated Hospital Sun Yat‐sen University Guangzhou Guangdong 510275 China; ^4^ Center of Hepato‐Pancreato‐Biliary Surgery the First Affiliated Hospital Sun Yat‐sen University Guangzhou Guangdong 510275 China; ^5^ Department of Breast and Thyroid Surgery Guangzhou Women and Children's Medical Center Guangzhou Medical University Guangzhou 510623 China

**Keywords:** breast cancer therapy, Copper/CTR1, NF‐κB, PD‐L1, TAK1

## Abstract

Copper plays a key role in inflammation and recent tumorigenesis. However, copper homeostasis and its role in cuproplasia and cuproptosis for cancer intervention remain incompletely explored. Here, it is unveiled that copper enhances the NF‐κB pathway by directly binding to transforming growth factor β‐activated kinase 1 (TAK1), thereby promoting TRAF2 interaction with and mediation of TAK1 ubiquitination and activation, leading to IκB kinase β (IKKβ) activation and mediating copper's inflammatory and oncogenic functions. Notably, copper is indispensable for TNFα/LPS‐induced NF‐κB activation and subsequent PD‐L1 promotion. Thus, copper chelators offer protection against acute infection in murine models. Meanwhile, NF‐κB represses copper uptake by negatively controlling the expression of copper transporter 1 (CTR1) transcriptionally, providing a negative feedback regulation for maintaining copper homeostasis. As a result, targeting NF‐κB appears to elevate CTR1 expression, leading to excessive copper uptake and downstream MAPK and AKT activation, in turn, conferring resistance to anti‐NF‐κB therapies. Therefore, disruption of NF‐κB not only synergizes with copper chelators to overcome drug resistance and cuproplasia, but also combines with copper ionophores to facilitate cuproptosis, providing a dual approach for combating chronic inflammation‐driven cancers.

## Graphical abstract

This study reveals a feedback mechanism regulating copper homeostasis, in which copper directly interacts with TAK1 to activate NF‐κB signaling, while NF‐κB transcriptionally suppresses copper transporter (CTR1) expression. These findings highlight a promising therapeutic strategy for breast cancer by combining NF‐κB inhibitors with copper chelators or cuproptosis inducers.

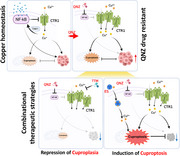

## 1 Introduction

Chronic inflammation serves as a major driver of tumorigenesis across various cancers, including breast cancer and hepatocellular carcinoma (HCC).^[^ ^1^, ^2^, ^3^, ^4^, ^5^ ^]^ Among that, NF‐κB pathway plays a central role in chronic inflammation triggered by cytokines like TNFα and IL‐6, as well as pathogen stresses such as LPS.^[^ ^6^, ^7^, ^8^, ^9^, ^10^ ^]^ Recently, environmental factor like lactate has also been identified to activate NF‐κB signaling pathway.^[^ ^11^ ^]^ However, the side‐effect of NF‐κΒ inhibitors in suppressing systemic immune responses limits their therapeutic potential in cancer treatment.^[^ ^12^, ^13^ ^]^ Thus, determining the upstream regulation of NF‐κΒ signaling and drug‐resistant mechanisms will offer a novel target and potential strategy for inflammation‐driven cancers.

Recent investigations have shed light on the intricate roles of copper in tumorigenesis. On one hand, besides influencing reactive oxygen species (ROS), elevated copper uptake can enhance tumorigenesis (termed cuproplasia) by directly binding and activating downstream oncogenic proteins such as MAPK, AKT and ULK1, to promote melanoma, NSCLC and breast cancer, respectively.^[^ ^14^, ^15^, ^16^ ^]^ However, other pathways involved in copper's oncogenic roles are still under investigation. On the other hand, excessive copper uptake including through the use of the copper ionophores, such as elesclomol (ES) and alcohol drug disulfiram (DSF), can induce a novel type of apoptosis (known as cuproptosis), in the presence of copper transporter 1 (CTR1),^[^ ^17^ ^]^ or Zn transporter 1 (ZnT1).^[^ ^18^ ^]^ Nevertheless, the mechanisms by which copper maintains homeostasis, and its regulation effects cuproplasia and cuproptosis for cancer therapies, remain largely elusive.

Although the potential role of copper chelator tetrathiomolybdate (TTM) in cancer therapy has been explored,^[^ ^19^, ^20^ ^]^ primarily benefiting patients with *CTR1* amplification,^[^ ^16^, ^21^, ^22^, ^23^, ^24^ ^]^ the stratification of patients for copper chelator therapy warrants further exploration, given the low rate of *CTR1* alterations. CTR1 expression is transcriptionally controlled by factors such as hypoxia‐inducible factor (HIF) or C‐Myc under different conditions.^[^ ^21^, ^25^ ^]^ Additionally, our recent study has also highlighted the regulation of CTR1 at a post‐translational level by AMPK‐mediated phosphorylation, suggesting the potential of combining AMPK agonists like metformin with copper chelators for breast cancer intervention.^[^ ^26^ ^]^ However, other upstream regulations of copper uptake, particularly those controlling CTR1 levels, remain poorly understood, which could provide potential promising strategies in synergizing with cuproptosis for cancer therapies.

In this study, we demonstrate that copper is essential for the pathophysiological activation of the NF‐κB pathway by directly binding to TAK1. Concurrently, activated NF‐κB negatively regulates *CTR1* expression to restrict copper uptake, thus establishing a negative feedback loop between the copper‐CTR1 and TAK1‐NF‐κB pathways. This highlights the potential strategy of combining NF‐κB inhibitors with copper chelators to inhibit cuproplasia or with copper ionophores to induce cuproptosis for breast cancer therapy.

## 2 Results

### 2.1 Copper‐CTR1 Axis Activates the NF‐κB Pathway to Perform Oncogenic Role

**Figure 1:**
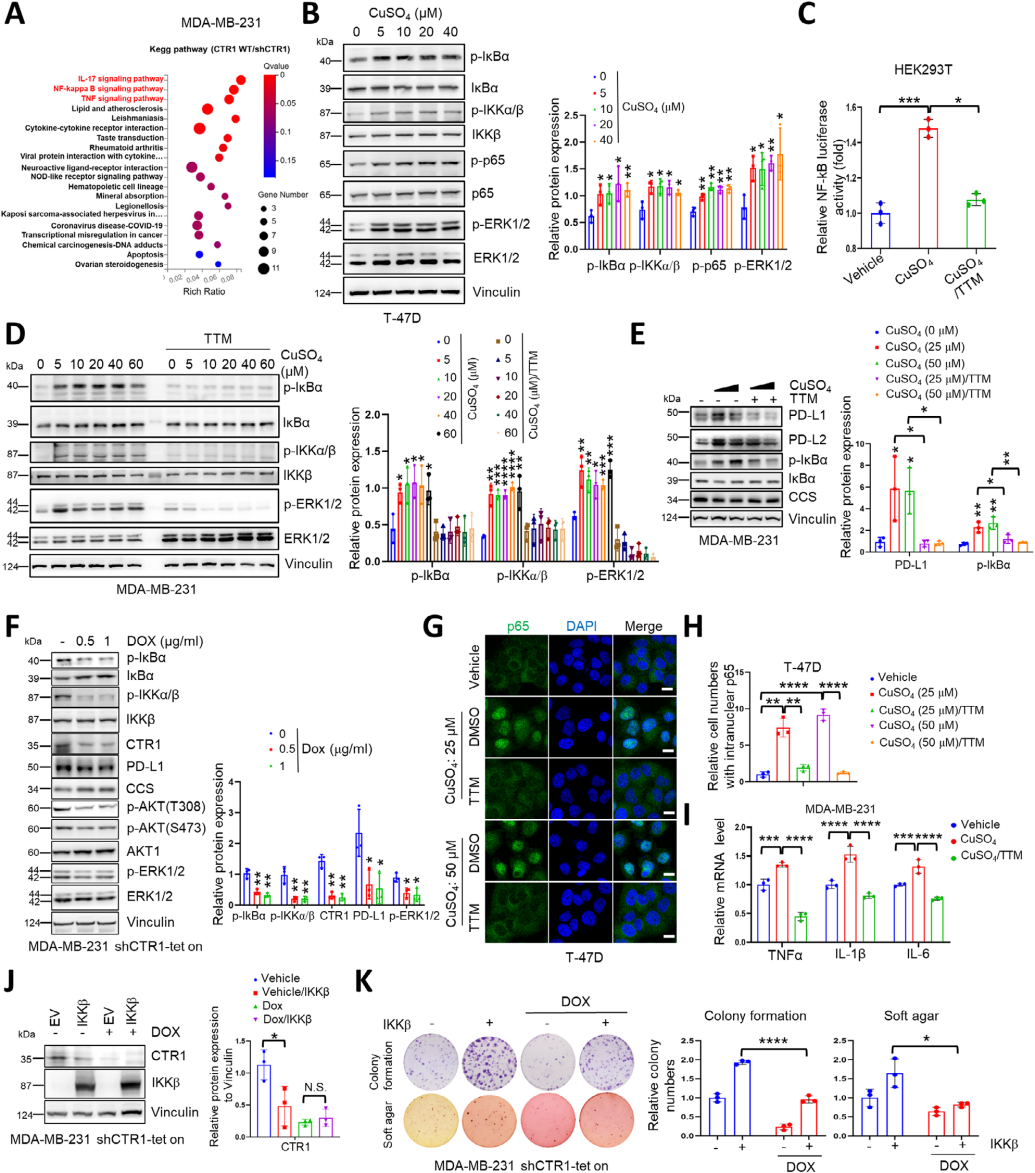
Copper activates NF‐κB pathway to mediate its oncogenic functions. A) MDA‐MB‐231 cells were infected with lentiviruses encoding shCTR1 and then selected with puromycin (1 µg mL^−1^). The resulting cells (MDA‐MB‐231 shCTR1‐tet on) were treated with or without doxycycline (1 µg mL^−1^) for 72 h and were collected for RNA extracting and sequencing (accession code: PRJCA027211). B) T‐47D cells were disposed with serum‐free medium and then treated with different concentrations of CuSO_4_ for indicated time point. The cells were collected and lysed for immunoblot (IB) analysis (left panel). The results were normalized and analyzed using student *t* test (mean+/‐SD, n = 3) (*p < 0.05, **p < 0.01, ***p < 0.001, and ****p < 0.0001) (right panel). C) HEK293T cells were transfected with NF‐κB luciferase reporter plasmid and then disposed with CuSO_4_ (50 µm) or/and TTM (50 µm) for 16 h. The cells were collected and lysed for the detection of luciferase activity. The results were normalized and analyzed using student *t* test (mean+/‐SD, n = 3) (*p < 0.05, **p < 0.01, ***p < 0.001, and ****p < 0.0001). D) MDA‐MB‐231 cells were cultured in serum‐free medium for 16 h. Then the cells were pretreated with TTM (100 µm) for 1 h and disposed with different concentrations of CuSO_4_ for 1 h. The resulting cells were subjected to IB analysis (left panel). The results were normalized and analyzed using student *t‐*test (mean+/‐SD, n = 3) (*p < 0.05, **p < 0.01, ***p < 0.001, and ****p < 0.0001) (right panel). E) MDA‐MB‐231 cells were cultured in serum‐free medium and treated with distinct concentrations of CuSO_4_ (25 µm, 50 µm) and TTM (50 µm) for 18 h and then subjected to IB analysis (left panel). The results were normalized and analyzed using student *t* test (mean+/‐SD, n = 3) (*p < 0.05, **p < 0.01, ***p < 0.001, and ****p < 0.0001) (right panel). F) MDA‐MB‐231 shCTR1‐tet on cells were treated with or without diverse concentrations of doxycycline for 72 h. Then the resulting cells were collected and lysed for IB analysis (left panel). The results were normalized and analyzed using student *t‐*test (mean+/‐SD, n = 3) (*p < 0.05, **p < 0.01, ***p < 0.001, and ****p < 0.0001) (right panel). G,H) T‐47D cells were disposed in serum‐free medium for 18 h and pretreated with TTM for 1 h, and then diverse concentrations of CuSO_4_ were added to the cells for another 2 h. The cells were subjected for immunofluorescence (IF) analysis (G). Bar indicates 10 µm. The cells with nuclear p65 were counted. The results were normalized and analyzed using student *t* test (mean+/‐SD, n = 3) (*p < 0.05, **p < 0.01, ***p < 0.001, and ****p < 0.0001) (H). I) MDA‐MB‐231 cells were treated in the presence or absence of CuSO_4_ (50 µm) or/and TTM (50 µm) for 16 h. The cells were collected for RNA extraction and then subjected to qRT‐PCR analysis. The results were analyzed using student *t* test (mean+/‐SD, n = 3) (*p < 0.05, **p < 0.01, ***p < 0.001, ****p < 0.0001). J,K) MDA‐MB‐231 shCTR1‐tet on cells were infected with lentiviruses encoding IKKβ and then selected with hygromycin (100 µg mL^−1^). The resulting cells were treated with or without doxycycline (1 µg mL^−1^) for 72 h and then subjected to IB analysis (J, left panel), colony formation (K, top panel) and soft agar assay (K, bottom panel). Relative protein expression (J, right panel) and relative colony numbers (K, right panel) were normalized and plotted. The results were analyzed using student *t* test (mean+/‐SD, n = 3) (*p < 0.05, **p < 0.01, ***p < 0.001, and ****p < 0.0001).

While the potential link between copper with infection and inflammation has been previously scratched,^[^ ^27^ ^]^ the underlying mechanism and clinical applications remain largely unclear. Through an RNA‐sequencing‐based approach, we observed that copper‐administration or depletion of its transporter *CTR1* closely associated with infection and inflammation, including the positive regulation of NF‐κB and TNFα pathways (**Figure** **1**A; Figure S1A, Supporting Information). Consistent with these findings, copper stimulation markedly increased NF‐κB activity, as evidenced by the phosphorylation of ΙκΒα and p65, in different cell lines in a dose‐dependent manner (Figure 1B; Figure S1B, Supporting Information). Similar results were also observed in NF‐κB luciferase reporter assays (Figure 1C). As expected, administration of the copper chelator TTM or depletion of copper transporter *CTR1* significantly reduced copper‐induced NF‐κB activation (Figure 1D–F; Figure S1C–G, Supporting Information). Of note, copper‐induced nuclear localization of p65 and the expressions of its downstream targeted genes such as *TNFα*, *IL‐6* and *IL‐1β* were attenuated by TTM treatment (Figure 1G–I).

To investigate whether copper mediates the oncogenic role of NF‐κB, we enforced expression of the central kinase of NF‐κB, IKKβ with or without the presence of CTR1 (Figure 1J). The results showed that IKKβ significantly elevated breast cancer cells colony formation, soft agar growth and cancer cell proliferation, which could be attenuated by depletion of *CTR1* (Figure 1K; Figure S1H, Supporting Information), indicating the potential roles of NF‐κB pathway in mediating copper oncogenic functions. These findings together suggest that copper plays crucial pathophysiological inflammatory roles by activating the NF‐κB pathway.

### 2.2 Copper is Essential for TNFα or LPS‐Induced NF‐κB Activation

**Figure 2:**
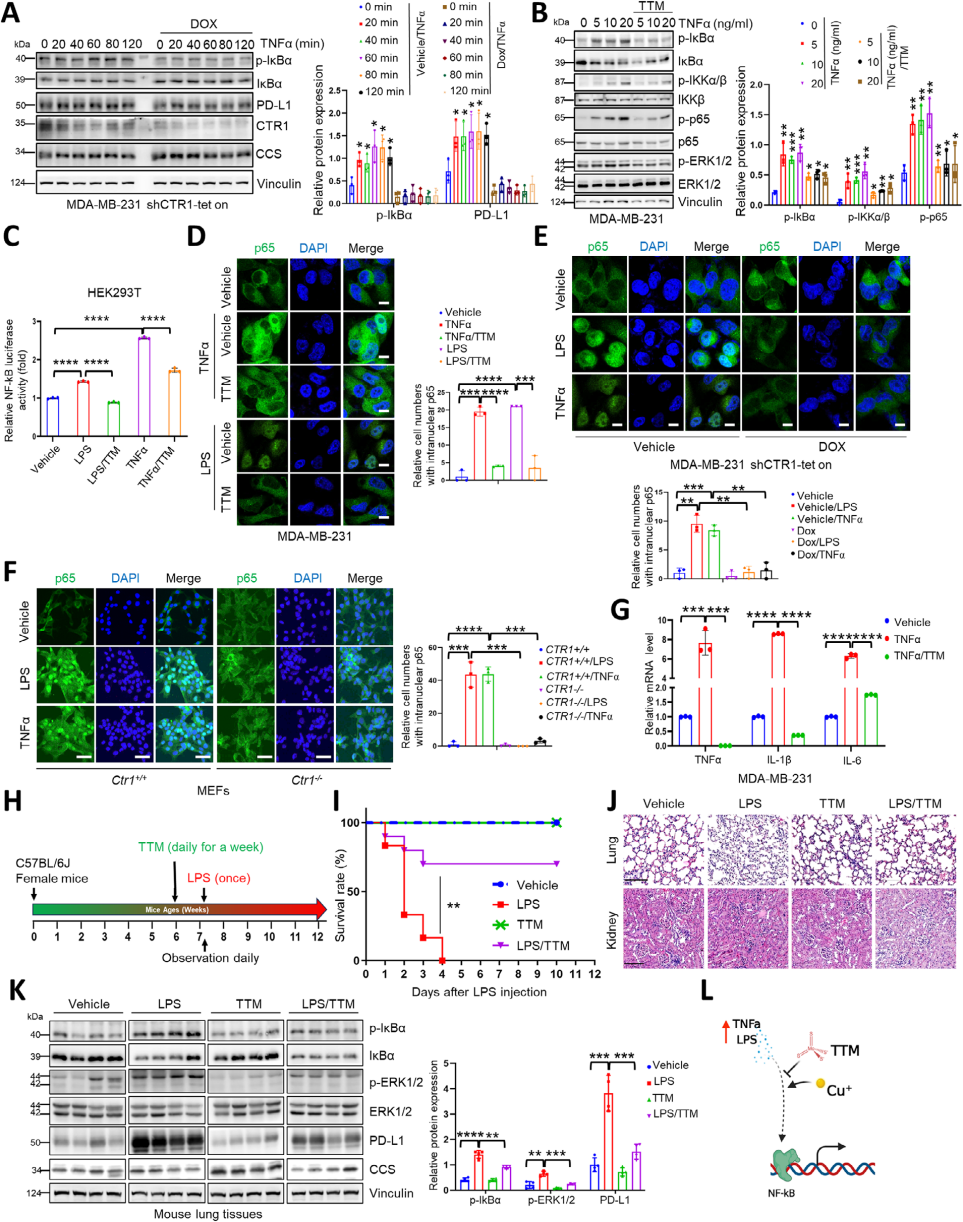
Copper is essential for TNFα/LPS‐induced NF‐κB activation. A) MDA‐MB‐231 shCTR1‐tet on cells were treated with or without doxycycline (1 µg mL^−1^) for 72 h and then the cells were disposed with TNFα for diverse time points. The resulting cells were subjected to IB analysis (left panel). The results were normalized and analyzed using student *t* test (mean+/‐SD, n = 3) (*p < 0.05, **p < 0.01, ***p < 0.001, and ****p < 0.0001) (right panel). B) MDA‐MB‐231 cells were pretreated with TTM (100 µM) for 1 h and disposed with distinct concentrations of TNFα for 1 h and then subjected to IB analysis (left panel). The results were normalized and analyzed using student *t‐*test (mean+/‐SD, n = 3) (*p < 0.05, **p < 0.01, ***p < 0.001, and ****p < 0.0001) (right panel). C) HEK293T cells were transfected with NF‐κB luciferase reporter plasmid and then disposed with TNFα (10 ng mL^−1^) or LPS (0.5 µg mL^−1^) in combination with or without TTM (50 µM) for 16 h. The cells were collected and lysed for the detection of luciferase activity. The results were normalized and analyzed using student *t‐*test (mean+/‐SD, n = 3) (*p < 0.05, **p < 0.01, ***p < 0.001, ****p < 0.0001). D) MDA‐MB‐231 cells were pretreated with TTM (100 µm) for 1 h and then disposed with TNFα (20 ng mL^−1^) and LPS (10 µg mL^−1^) for 1 h, respectively. The cells were fixed, permeated and subjected for IF staining (left panel). Bar indicates 10 µm. The cells with nuclear p65 were counted. The results were normalized and analyzed using student *t* test (mean+/‐SD, n = 3) (*p < 0.05, **p < 0.01, ***p < 0.001, and ****p < 0.0001) (right panel). E) MDA‐MB‐231 shCTR1‐tet on cells were treated with or without doxycycline (1 µg mL^−1^) for 72 h and then treated with TNFα (20 ng mL^−1^) and LPS (10 µg mL^−1^) for 1 h, respectively. The cells were fixed, permeated and subjected for IF staining (top panel). Bar indicates 10 µm. The cells with nuclear p65 were counted. The results were normalized and analyzed using student *t* test (mean+/‐SD, n = 3) (*p < 0.05, **p < 0.01, ***p < 0.001, and ****p < 0.0001) (bottom panel). F) *Ctr1^‐/‐^* and counterpart MEFs cells were treated with TNFα (50 ng mL^−1^) and LPS (10 µg mL^−1^) for 1 h, respectively. The resulting cells were fixed, permeated, and subjected for IF staining (left panel). Bar indicates 50 µm. The cells with nuclear p65 were counted. The results were normalized and analyzed using student *t* test (mean+/‐SD, n = 3) (*p < 0.05, **p < 0.01, ***p < 0.001, and ****p < 0.0001) (right panel). G) MDA‐MB‐231 cells were treated with TNFα (10 ng mL^−1^) in the presence or absence of TTM (50 µM) for 15 h. The cells were collected for RNA extraction and then subjected to qRT‐PCR analyses. The results were analyzed using student *t* test (mean+/‐SD, n = 3) (*p < 0.05, **p < 0.01, ***p < 0.001, and ****p < 0.0001). H) The time schematic of the female C57BL/6 mice disposed with LPS or/and TTM. I–K) The female C57BL/6 mice (6/each group) were pretreated with indicated concentration of TTM and then disposed with LPS (10 mg kg^−1^) or/and TTM. After 6 h, the lung tissues were collected and utilized for H&E staining (J) and IB analyses (K, left panel). Relative protein expression was normalized and plotted (K, right panel). Survivals of the mice were recorded every day until no mice lived in the group of treated with LPS alone. Survival curve was drawn by GraphPad Prism version 6.0 (I) and the results were analyzed using Log‐rank (Mantel‐Cox) test (**p < 0.01). Control group (blue), LPS group (red), TTM group (green), LPS/TTM group (violet). Bar indicates 100 µm. The results in (K) were analyzed using student *t* test (mean+/‐SD, n = 3) (*p < 0.05, **p < 0.01, ***p < 0.001, and ****p < 0.0001). L) A proposed model for TNFα or LPS‐induced activation of NF‐κB pathway mediated by copper. Copper has a key role in TNFα or LPS‐induced activation of NF‐κB pathway, whereas, copper chelator TTM suppresses activation of NF‐κB pathway induce by TNFα or LPS.

Given that TNFα, LPS and virus infection are canonical factors for activation of NF‐κB pathway, we sought to investigate whether copper could affect these canonical upstream regulators for NF‐κB signaling. Interestingly, depletion of *CTR1* largely attenuated both TNFα and LPS‐induced NF‐κB activation in breast cancer cells and mouse embryonic fibroblasts (MEFs) (**Figure** **2**A; Figure S2A–C, Supporting Information). Consistently, TNFα‐ and LPS‐ or enforced expression of IKKα/β‐induced NF‐κB activation was readily repressed by TTM administration (Figure 2B,C; Figure S2D–G, Supporting Information). Moreover, p65 nuclear localization and its downstream target genes such as *TNFα*, *IL‐6* and *IL‐1* *β* were strongly blocked upon TTM treatment or depletion of *CTR1* (Figure 2D–G; Figure S2H,I, Supporting Information). Biologically, in an LPS‐based acute infection mouse model, TTM markedly decreased LPS‐induced NF‐κB activation and acute injuries to the lung and kidney, facilitating mouse survival (Figure 2H–K; Figure S2J, Supporting Information). These findings suggest that the copper‐CTR1 axis is essential for canonical cytokines or pathogens‐induced NF‐κB activation (Figure 2L).

### 2.3 Copper‐CTR1 Elevates PD‐L1 Expression via Activating the NF‐κB Pathway

Next, based on a previous reported connection of copper with PD‐L1 expression,^[^ ^22^ ^]^ we investigated whether and how copper regulates PD‐L1. To this end, we administrated different inhibitors to block copper‐regulated pathways including MAPK, AKT, and NF‐κB, and observed that these inhibitors could potentially attenuate copper‐induced PD‐L1 expression, accompanied by repression of NF‐κB signaling, a well‐established upstream regulator of PD‐L1 (Figure S3A, Supporting Information). Thus, we observed that copper‐induced activation of NF‐κB played a major role in boosting PD‐L1 expression in vitro and in vivo, accompanied by increased CD8^+^ cells in mammary tumors derived from MMTV‐PyMT mice, which was notably reversed by the treatment of copper chelator TTM or depletion of *CTR1* (Figure 1E,F; Figure S3B–F, Supporting Information). In line with this finding, TTM or depletion of *CTR1* could abolish TNFα or LPS‐induced PD‐L1 expression via repressing the NF‐κB pathway (Figure 2A; Figures S2A and S3F–I, Supporting Information). Similar results were also observed in LPS‐induced mouse model that TTM could efficiently decrease PD‐L1 expression in different tissues (Figure 2K; Figure S2J, Supporting Information). Interestingly, *CTR1* expression was positively correlated with *PD‐L1* expression at the transcriptional levels in various types of cancers, including breast cancer (Figure S3J, Supporting Information). Meanwhile, we observed the tight positive correlation of CTR1 and PD‐L1 expression in breast cancer tissues with lower expression of both in adjacent normal tissues (Figure S4A, Supporting Information). In echoing this finding, we employed a breast syngeneic mouse model with murine mammary cancer E0771 cells treated with TTM. The result showed that CD8^+^ cells were largely increased, accompanied by reducing PD‐L1 expression (Figure S4B, Supporting Information), suggesting the potential role of copper in promoting NF‐κB activating and its downstream PD‐L1 expression, which conferred cancer cell immune evasion. Next, we employed an *ex vivo* T cell‐based cytotoxic assay, and observed that pretreated breast cancer cells with TTM or bearing *CTR1* depletion could promote T cell‐mediated tumor killing effect (Figure S4C, Supporting Information). These findings together demonstrate that the copper‐CTR1 axis regulates tumor immune response to promote tumor immune evasion in part via activating NF‐κB‐mediated PD‐L1 expression.

### 2.4 Copper Directly Binds TAK1 to Activate IKKβ‐Mediated NF‐κB Signaling

Although a previous report shows that copper could regulate XIAP to affect NF‐κB signaling,^[^ ^28^ ^]^ here, in *XIAP*‐depleted cells, we still found copper‐mediated activation of NF‐κB signaling (Figure S5A, Supporting Information). Thus, we sought to explore the potential mechanism of copper in activating the NF‐κB pathway. For that, copper enhanced the phosphorylation of IκBα, a negative regulator of the NF‐κB pathway, in cells, accompanied by readily enhanced IKKβ phosphorylation (Figure 1), without effect on the complex formation of IKKα/IKKβ/NEMO (Figure S5B, Supporting Information). Due to the potential of copper to bind with and activate kinases, such as MEK, ULK, and PDK1,^[^ ^14^, ^15^, ^16^ ^]^ we further investigated whether copper could directly bind and activate IKKβ, the central kinase of canonical NF‐kB signaling pathway. To this end, IKKβ in vitro kinase assays showed that IKKβ, derived from copper‐pretreated cells, but not directly adding copper into the kinase reactions, exhibited elevated kinase activity, which could be blocked by administration of TTM (Figure S5C,D, Supporting Information), indicating that copper regulates the upstream regulators of IKKβ instead of directly enhancing IKKβ kinase activity.

**Figure 3:**
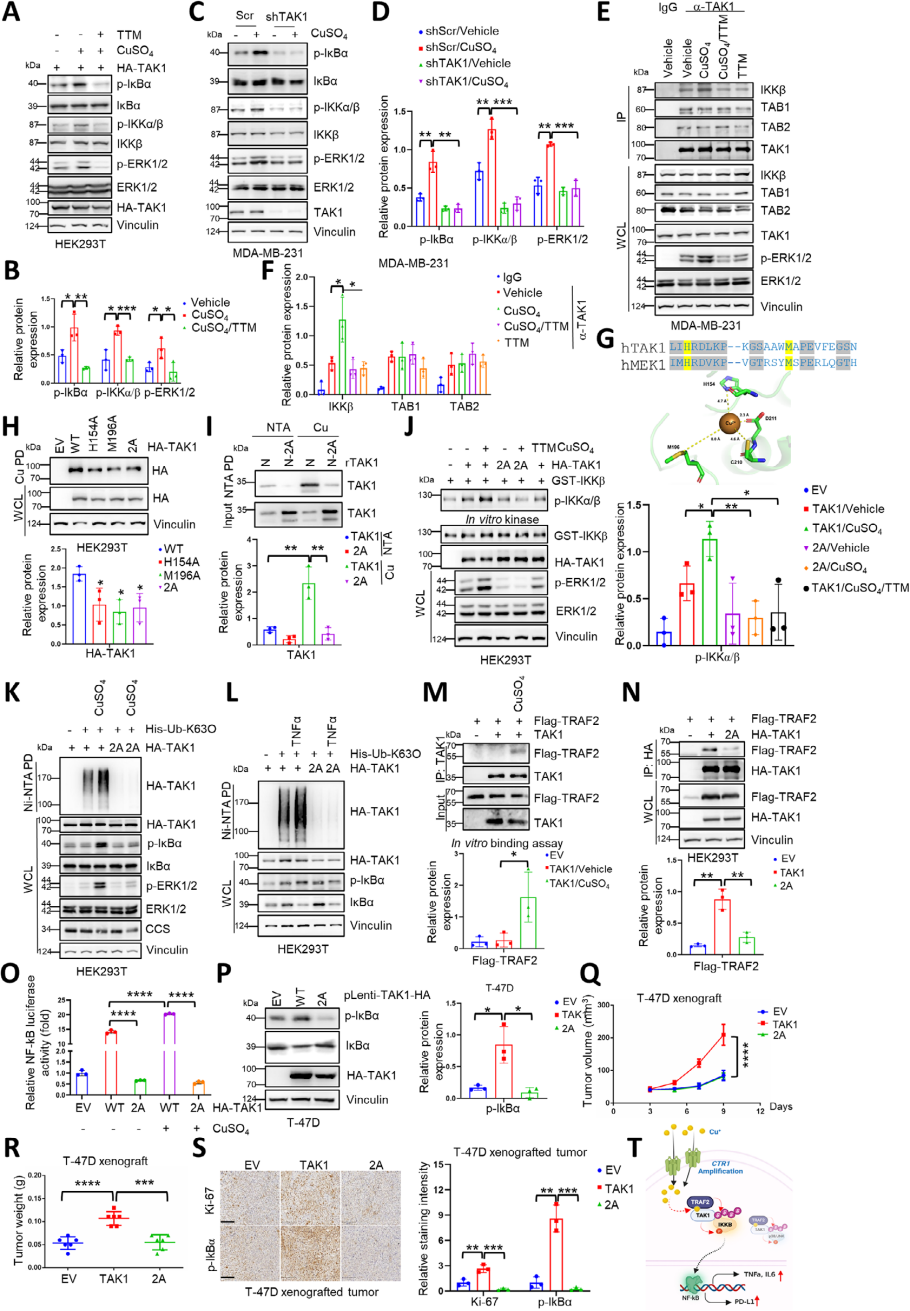
Copper activates NF‐κB pathway through binding with and activating TAK1‐mediated IKKβ phosphorylation. A,B) HEK293T cells were transfected with indicated construct and then were treated with CuSO_4_ (50 µm) and TTM (100 µm) for 1 h after disposed in serum‐free medium for 16 h. The resulting cells were collected and subjected to IB analysis (A). The results were normalized and analyzed using student *t* test (mean+/‐SD, n = 3) (*p < 0.05, **p < 0.01, ***p < 0.001, and ****p < 0.0001) (B). C,D) MDA‐MB‐231 were infected with lentiviruses encoding shTAK1 and then selected with puromycin (1 µg mL^−1^). The resulting cells and control cells were disposed in serum‐free medium for 16 h and then treated with CuSO_4_ (100 µM) for 1 h. The cells were collected and subjected to IB analysis (C). Relative protein expression was normalized and plotted (D). The results were analyzed using student *t* test (mean+/‐SD, n = 3) (*p < 0.05, **p < 0.01, ***p < 0.001, and ****p < 0.0001). E,F) MDA‐MB‐231 cells were disposed in serum‐free medium and treated with CuSO_4_ (50 µm) or/and TTM (50 µm) for 14 h. The resulting cells were collected and lysed. The lysate was incubated with anti‐TAK1 antibody and protein A/G agarose for 12 h a 4 °C. The agaroses were washed with NETN buffer (20 mm Tris‐HCl (PH 8.0), 150 mm NaCl, 0.5% NP‐40, 1 mm EDTA) for 4 times and then subjected to IB analysis (E). Relative protein expression was normalized and plotted (F). The results were analyzed using student *t* test (mean+/‐SD, n = 3) (*p < 0.05, **p < 0.01, ***p < 0.001, and ****p < 0.0001). G) The amino acid sequence of human MEK1 and TAK1 was aligned using Clustal W. Blue letters: amino acids. Blue letters in yellow background represented copper‐binding conserved sites between MEK1 and TAK1. Spatial structure and the intervening space (Å) of interaction of TAK1 containing amino acids H154 and M196 with copper were performed by Schrodinger software package. H) HEK293T cells were transfected with indicated constructs, resulting cells were harvested for copper pull‐down and then subjected to IB analysis (top panel). The results were normalized and analyzed using student *t* test (mean+/‐SD, n = 3) (*p < 0.05, **p < 0.01, ***p < 0.001, and ****p < 0.0001) (bottom panel). I) The fragment TAK1‐N containing 300 amino acids derived from N terminal of human TAK1 and the mutant fragment TAK1‐N‐2A containing H154A and M196A sites were inserted into the vector pGEX‐4T1. The proteins TAK1‐N and TAK1‐N‐2A were purified from bacteria (*E. coli* strain BL21). The GST tag was cleaved using Thrombin. The resulting protein was incubated with NTA beads with or without copper for 0.5 h at 4 °C. The beads were washed using NETN buffer (20 mm Tris‐HCl (PH 8.0), 300 mm NaCl, 0.5% NP‐40, 1 mm EDTA) for 5 times and then subjected to IB analysis (top panel). Relative protein expression was normalized and plotted (bottom panel). The results were analyzed using student *t* test (mean+/‐SD, n = 3) (*p < 0.05, **p < 0.01, ***p < 0.001, and ****p < 0.0001). J) HEK293T cells were transfected with indicated constructs and then disposed with CuSO_4_ (100 µm) individually or in combination with TTM (100 µm) for 40 mins after cultivated in serum‐free medium for 16 h, following the kinase TAK1 was immuno‐precipitated from cells above. The substrate IKKβ was immuno‐precipitated and purified from HEK293T transfected with indicated constructs. The indicated purified TAK1 and IKKβ were used for kinase reaction and then subjected to IB analysis (top panel). The results were normalized and analyzed using student *t* test (mean+/‐SD, n = 3) (*p < 0.05, **p < 0.01, ***p < 0.001, and ****p < 0.0001) (bottom panel). K,L) HEK293T cells were cotransfected with indicated constructs. Following, the cells were disposed with CuSO_4_ (50 µm) in the serum‐free medium (K) or treated with TNFα (10 ng mL^−1^) (L) for 10 h. The cells were collected and lysed. The lysate was incubated with Ni‐NTA beads for 3 h at room temperature. The beads were washed and then subjected to IB analysis. M) HEK293T cells were transfected with the plasmid Flag‐TRAF2. The resulting cells were harvested and lysed. The cell lysate was incubated with anti‐Flag beads for 3 h at 4 °C and then the beads were washed using NETN buffer (20 mm Tris‐HCl (PH 8.0), 150 mm NaCl, 0.5% NP‐40, 1 mm EDTA) for 4 times. Following, the protein Flag‐TRAF2 was replaced from the beads using 3 x Flag peptide. The purified Flag‐TRAF2 and commercialized TAK1 were co‐incubated with anti‐TAK1 antibody and protein A/G agarose with or without CuSO_4_ (50 µM) for 10 h at 4 °C and then the beads were washed using NETN buffer for 4 times and subjected to IB analysis (top panel). The results were normalized and analyzed using student *t* test (mean+/‐SD, n = 3) (*p < 0.05, **p < 0.01, ***p < 0.001, and ****p < 0.0001) (bottom panel). N) HEK293T cells were cotransfected with indicated constructs. The resulting cells were harvested and subjected to immunoprecipitation (IP) with anti‐HA beads and IB analysis (top panel). The results were normalized and analyzed using student *t‐*test (mean+/‐SD, n = 3) (*p < 0.05, **p < 0.01, ***p < 0.001, and ****p < 0.0001) (bottom panel). O) HEK293T cells were transfected with NF‐κB luciferase reporter plasmid and indicated constructs. Following, the cells above were disposed with CuSO_4_ (50 µm) for 16 h. The cells were collected and lysed for the detection of luciferase activity. The results were analyzed using student *t* test (mean+/‐SD, n = 3) (*p < 0.05, **p < 0.01, *** *p* < 0.001, ****p < 0.0001). P) T‐47D cells stably expressing wild‐type or mutant TAK1 were harvested and subject to IB analysis (left panel). The results were normalized and analyzed using student *t* test (mean+/‐SD, n = 3) (*p < 0.05, **p < 0.01, ***p < 0.001, and ****p < 0.0001) (right panel). Q–S) T‐47D cells expressing wild‐type or mutant TAK1 were subjected to xenograft assays. The tumor size was monitored (mean+/‐SD, n = 6) (****p < 0.0001, *ANOVA* test) (Q). The tumors were dissected and weighed (R) (mean+/‐SD, n = 6). (***p < 0.001, student *t* test). The tumors were subjected to IHC assay with indicated antibodies (S, left panel). Bar indicates 100 µm. The staining intensity was normalized, plotted (S, right panel), and analyzed (mean+/‐SD, n = 3) (**p < 0.01, ***p < 0.001, student *t* test). T) A proposed model for the activation of NF‐κB pathway by copper. Under *CTR1* amplification, excess copper directly binds to TAK1 and strengthens the E3 ubiquitin ligase TRAF2 to bind with TAK1 to facilitate K63‐linked ubiquitination of TAK1, resulting in activation of the kinases TAK1 and IKKβ and subsequent activation of NF‐κB pathway to facilitate cytokines (TNFα, IL‐6) and PD‐L1 expressions.

TAK1 is a well‐established upstream kinase of IKKβ, which is a common downstream effector of TNFα and LPS in mediated NF‐κΒ signaling.^[^ ^29^, ^30^ ^]^ Thus, we explored whether copper could positively regulate TAK1 in phosphorylating IKKβ. Interestingly, TAK1‐mediated IKKβ activation was promoted by copper and blocked by TTM both in cells and in vitro (**Figure** **3**A,B,J). Moreover, pharmacological inhibition or genetic depletion of *TAK1* blocked copper‐induced p‐IKKβ and NF‐κB activation (Figure 3C,D; Figure S5E,F, Supporting Information). Further studies evaluated that copper could interact with TAK1 both in cells and in vitro in the kinase domain (Figure S5G–I, Supporting Information), thus enhancing TAK1 interaction with and phosphorylation of IKKβ (Figure 3E,F; Figure S5J, Supporting Information).

Due to the capability of copper in directly binding to kinases, in particular MEK,^[^ ^14^ ^]^ we aligned the amino acids of TAK1 with MEK1 for the kinase domain, a similar approach previously used in identification of copper binding sites in ULK1.^[^ ^15^ ^]^ As such, two resides of histidine and methionine in TAK1 (H154 and M196) have been virtually docked with MEK1 as copper potential binding residues (Figure 3G). Next, we generated the non‐binding mutants (H154A and/or M196A), and observed that the interaction of copper with mutant TAK1 was markedly reduced compared with intact species both in cell and in vitro (Figure 3H,I). Meanwhile, the copper‐induced interaction between TAK1 and IKKβ, but not TAB1 or TAB2, was significantly abolished by TTM or in copper binding‐deficient TAK1 (Figure 3E,F; Figure S5J–L, Supporting Information). Interestingly, although copper induced TAK1 phosphorylating IKKβ in cells, it did not repress TAK1 kinase activity once added in tube for kinase reaction (Figure S5M, Supporting Information), suggesting that the binding of copper does not affect TAK1 kinase activity in vitro.

### 2.5 Copper Binds TAK1 to Enhance TRAF2‐Mediated Interaction with and Ubiquitination of TAK1 to Activate NF‐κB Pathway

Ubiquitin modification plays a predominant role in NF‐kB activation, in particular by tumor necrosis factor receptor‐associated factors (TRAFs)‐mediated K63‐linked ubiquitin.^[^ ^31^ ^]^ Heavily ubiquitin modification of TAK1 has been characterized for its interaction with upstream regulator and downstream effectors. Thus, here we detected whether copper could influence TAK1 ubiquitination. The results showed that copper markedly enhanced TAK1 K63‐linked ubiquitination, while copper‐binding‐deficient mutant (2A) blocked copper‐induced TAK1 ubiquitination (Figure 3K; Figure S5N, Supporting Information). Interestingly, in line with the essential role of copper in TNFα‐mediated activation of NF‐κB, TNFα elevated K63‐linked ubiquitination in WT, but not in mutant TAK1 (2A) (Figure 3L; Figure S5O, Supporting Information), indicating the potential role of copper in promoting TAK1 ubiquitin modification. Moreover, we detected the well‐established E3 ligase for TAK1, and observed that, copper enhanced TAK1 interaction with TRAF2, but not TRAF6, which could be readily abolished by TTM administration (Figure S5P, Supporting Information). Furthermore, copper could directly enhance TAK1 interaction with TRAF2 in vitro (Figure 3M; Figure S5P, Supporting Information), while copper‐binding deficient mutant blocked their interaction (Figure 3N; Figure S5Q,R, Supporting Information). These findings together suggest that copper binds TAK1 to recruit TRAF2 and promote TAK1 ubiquitination, resulting in TAK1 binding IKKβ and activating NF‐κB pathway.

Next, we detected the effect of 2A‐TAK1, and observed that these mutants not only block copper‐induced NF‐κB activation, but also repressed TNFα‐induced NF‐κB activation (Figure 3O; Figure S6A–D, Supporting Information). We further generated the cells harboring different TAK1 mutants, and observed that mutant TAK1 (HM to AA) decreased its capability to phosphorylate IKKβ to activate the NF‐κB pathway upon copper or TNFα stimulation (Figure S6E–I, Supporting Information). As a result, these TAK1 mutants defected TAK1's oncogenic functions to promote cancer cell proliferation, tumor growth in vivo, and elevate p65 downstream targeted genes such as *TNFα*, *IL‐6* and *IL‐1β*. (Figure 3P–S; Figure S6J–O, Supporting Information). Therefore, these findings together suggest that TAK1 is a binding partner of copper to mediate copper‐induced IKKβ‐NF‐κB activation (Figure 3T).

### 2.6 NF‐κB Negatively Regulates CTR1 Expression

**Figure 4:**
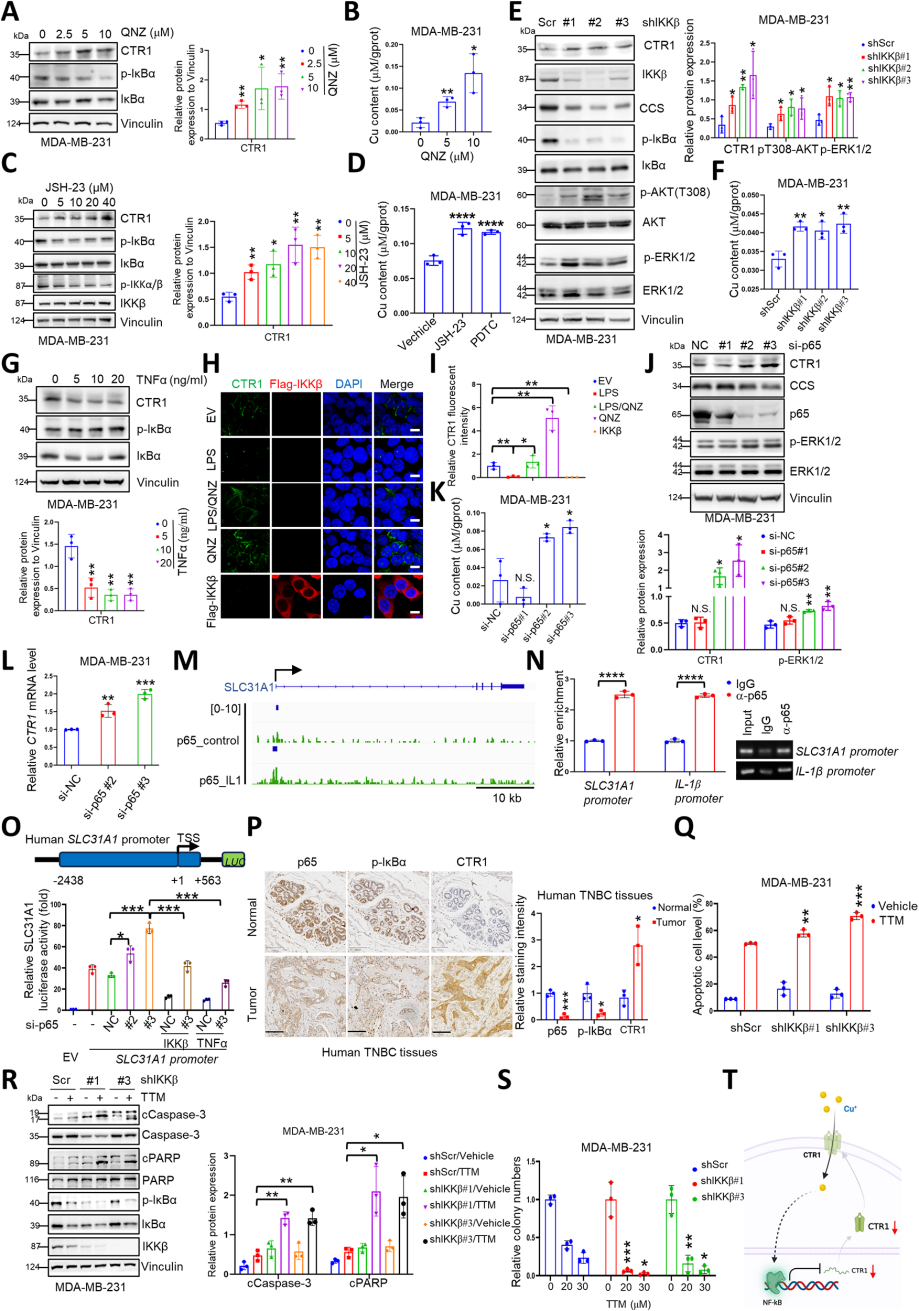
NF‐κB transcriptionally represses *CTR1* expression. A) MDA‐MB‐231 cells were treated with different concentrations of QNZ for 14 h. The resulting cells were collected and subjected to IB analysis (left panel). The results were normalized and analyzed using student *t‐*test (mean+/‐SD, n = 3) (*p < 0.05, **p < 0.01) (right panel). B) MDA‐MB‐231 cells were disposed with different concentrations of QNZ for 14 h and then collected for measuring the copper content. The results were normalized and analyzed using student *t* test (mean+/‐SD, n = 3) (*p < 0.05, ** *p* < 0.01). C) MDA‐MB‐231 cells were treated with different concentrations of NF‐κB inhibitor JSH‐23 for 14 h. The resulting cells were collected and subjected to IB analysis (left panel). The results were normalized and analyzed using student *t‐*test (mean+/‐SD, n = 3) (*p < 0.05, **p < 0.01) (right panel). D) MDA‐MB‐231 cells were disposed with NF‐κB inhibitor JSH‐23 (20 µm) or PDTC (1 µM) for 14 h and then collected for measuring the copper content. The results were normalized and analyzed using student *t‐*test (mean+/‐SD, n = 3) (****p < 0.0001). E) MDA‐MB‐231 cells were infected with shRNA lentiviruses targeting *IKKβ* and then selected with puromycin (1 µg mL^−1^). The resulting cells were collected and subjected to IB analysis (left panel). The results were normalized and analyzed using student *t* test (mean+/‐SD, n = 3) (*p < 0.05, **p < 0.01, ****p < 0.0001) (right panel). F) The cells from (E) were collected for measuring the copper content. The results were normalized and analyzed using student *t* test (mean+/‐SD, n = 3) (*p < 0.05, **p < 0.01). G) MDA‐MB‐231 cells were treated with different concentrations of TNFα for 14 h. The resulting cells were collected and subjected to IB analysis (top panel). The results were normalized and analyzed using student *t‐*test (mean+/‐SD, n = 3) (**p < 0.01) (bottom panel). (H‐I) HEK293T cells were transfected with indicated construct or disposed with LPS (1 µg mL^−1^) or/and QNZ (10 µM) for 14 h. The resulting cells were fixed, permeated and stained (H). Bar indicates 10 µm. The fluorescent intensity of CTR1 was measured and plotted (I). The results were analyzed using student *t‐*test (mean+/‐SD, n = 3) (*p < 0.05, **p < 0.01). J) MDA‐MB‐231 cells were transfected with small interfering RNAs targeting *p65* for 72 h. The resulting cells were collected and subjected to IB analysis (top panel). The results were normalized and analyzed using student *t* test (mean+/‐SD, n = 3) (*p < 0.05, N.S., no significance) (bottom panel). K) MDA‐MB‐231 cells were transfected with small interfering RNAs targeting *p65* for 72 h. Then the cells were collected for measuring the copper content. The results were normalized and analyzed using student *t* test (mean+/‐SD, n = 3) (*p < 0.05, N.S., no significance). L) MDA‐MB‐231 cells were transfected with small interfering RNAs targeting *p65* for 48 h. The resulting cells were collected for RNA extraction and then subjected to qRT‐PCR analysis. The results were analyzed using student *t* test (mean+/‐SD, n = 3) (**p < 0.01, ***p < 0.001). M,N) The binding affinity of p65‐*SLC31A1* promoter with or without IL‐1β stimulation was acquired from GEO database using the IGV (version 2.17.0) genome browser. Green indicated *SLC31A1* gene tracks and blue indicated annotation tracks (M). ChIP‐qPCR analyses of the enrichment of *SLC31A1* promoter and *IL‐1β* promoter by p65 (N, left panel) and the products were identified by DNA gel electrophoresis (N, right panel). The results were analyzed using student *t* test (mean+/‐SD, n = 3) (****p < 0.0001). O) Construct of the luciferase reporter plasmid containing human *SLC31A1* promoter. The promoter sequences were located at between the upstream 2438 locus and the downstream 563 locus from transcription start site (TSS) (top panel). HEK293T cells were transfected with small interfering RNAs targeting *p65* for 24 h and then transfected with the luciferase reporter plasmid containing human *SLC31A1* promoter or/and the indicated constructs. After 36 h of transfection, the cells were treated with or without TNFα (5 ng mL^−1^) for 16 h. The resulting cells were collected and lysed for the detection of luciferase activity (bottom panel). The results were analyzed using student *t‐*test (mean+/‐SD, n = 3) (*p < 0.05, ***p < 0.001). P) IHC analyses of human TNBC tissues and normal breast tissues with indicated antibodies (left panel). Bar indicates 100 µm. The staining intensity was normalized and plotted (right panel). The results were analyzed using student *t* test (mean+/‐SD, n = 3) (*p < 0.05, ***p < 0.001). Q–S) *IKKβ* knock‐down MDA‐MB‐231 and control cells were disposed with different concentrations of TTM (Q‐R, 60 µm; S, 20 µm or 30 µm) for 72 h (apoptosis assay) or 14 days (colony formation) and then subjected to apoptosis assay (Q), IB analysis (R, left panel), and colony formation (S). Apoptotic cells, relative colony numbers and relative protein expression (R, right panel) were normalized and plotted. The results were analyzed using student *t* test (mean+/‐SD, n = 3) (*p < 0.05, **p < 0.01, ***p < 0.001). T) A proposed model for copper‐induced NF‐κB activation to suppress CTR1 transcriptional expression and subsequently reducing cellular copper level.

Although the regulation of *CTR1* by C‐Myc and HIF transcriptionally,^[^ ^21^, ^25^ ^]^ as well as by AMPK and Nedd4l post‐translationally,^[^ ^26^ ^]^ has been reported, whether NF‐κB could regulate CTR1 expression and modulate copper uptake is not yet defined. With the administration of NF‐κB inhibitors, we observed a significant increase in CTR1 protein levels and copper uptake (**Figure** **4**A–D; Figure S7A–C, Supporting Information), accompanied by increased *CTR1* mRNA (Figure S7D,E, Supporting Information). Furthermore, repression of the NF‐κB pathway by genetic deletion of *IKKβ* or enforced expression of negative form IκBα (DN), enhanced CTR1 expression and copper uptake (Figure 4E,F; Figure S7F–I, Supporting Information), accompanied by increased *CTR1* mRNA levels (Figure S7J, Supporting Information). Conversely, activation of the NF‐κB pathway with TNFα or LPS, or ectopic expression of IKKβ, markedly reduced CTR1 expression and membrane localization (Figure 4G–I; Figure S7K, Supporting Information), while NF‐κB inhibitors restored TNFα or LPS‐reduced CTR1 expression and membrane localization (Figure 4H,I; Figure S7L,M, Supporting Information). Simultaneously, ectopic expressing IKKβ‐mediated reduction of CTR1 expression could not be restrained by proteasomal or autophagy inhibitors (Figure S7N, Supporting Information). These findings indicate a negative regulation of CTR1 by the NF‐κB pathway at the transcriptional level.

To uncover the underlying mechanism of NF‐κB in regulating CTR1 expression, we observed that NF‐κB repressed *CTR1* expression at the transcriptional level (Figure S7O,P, Supporting Information), and depletion of *p65*, the canonical NF‐κB transcriptional factor, markedly increased CTR1 expression both in protein and mRNA levels, as well as elevated copper uptake (Figure 4J–L; Figure S7Q, Supporting Information). Thus, we hypothesized that p65 acted as the transcriptional factor to negatively regulate *CTR1* expression. To assess whether p65 directly regulated *CTR1* expression, we analyzed previously established anti‐p65‐based ChIP‐sequencing results,^[^ ^32^ ^]^ and found that indeed p65 was observed directly binding to the promoter region of *CTR1* (*SLC31A1*), with the binding affinity further increased upon IL‐1β stimulation (Figure 4M). Next, the binding of p65 with the *SLC31A1* promoter was further validated by ChIP‐qPCR, with *IL‐1* *β* promoter as a positive control (Figure 4N). Additionally, depletion of *p65* significantly repressed the altered transcriptional activity of *SLC31A1* induced by IKKβ expression or TNFα stimulation compared to enforcing IκBα‐DN expression or QNZ administration (Figure 4O; Figure S7R, Supporting Information). In line with this, we observed a reserve correlation of p65 activation (p‐p65 or nuclear p65) or IκBα phosphorylation with CTR1 expression in breast cancer cells (Figure S7S, Supporting Information), and breast cancer tissues (Figure 4P), indicating a potential negative regulation of CTR1 by the NF‐κB pathway. Next, we disrupted the copper homeostasis by depleting *IKKβ* to upregulate CTR1‐copper axis, and observed that cells with *IKKβ* deficiency exhibited more sensitivity to TTM administration, measured with cell apoptosis (Figure 4Q; Figure S7T, Supporting Information) and apoptotic markers, such as cleaved Capspase‐3 (cCaspase‐3) and cleaved PARP (cPARP) (Figure 4R), or with colony formation assays (Figure 4S; Figure S7U, Supporting Information). Therefore, these findings offer a mechanism of CTR1 being negatively regulated by TNFα/LPS‐induced NF‐κB activation and unravel a negative feedback regulation of CTR1 via copper‐NF‐κB pathway (Figure 4T).

### 2.7 Activating the Copper‐CTR1 Axis Contributes to the Resistance of Targeting NF‐kB Signaling

**Figure 5:**
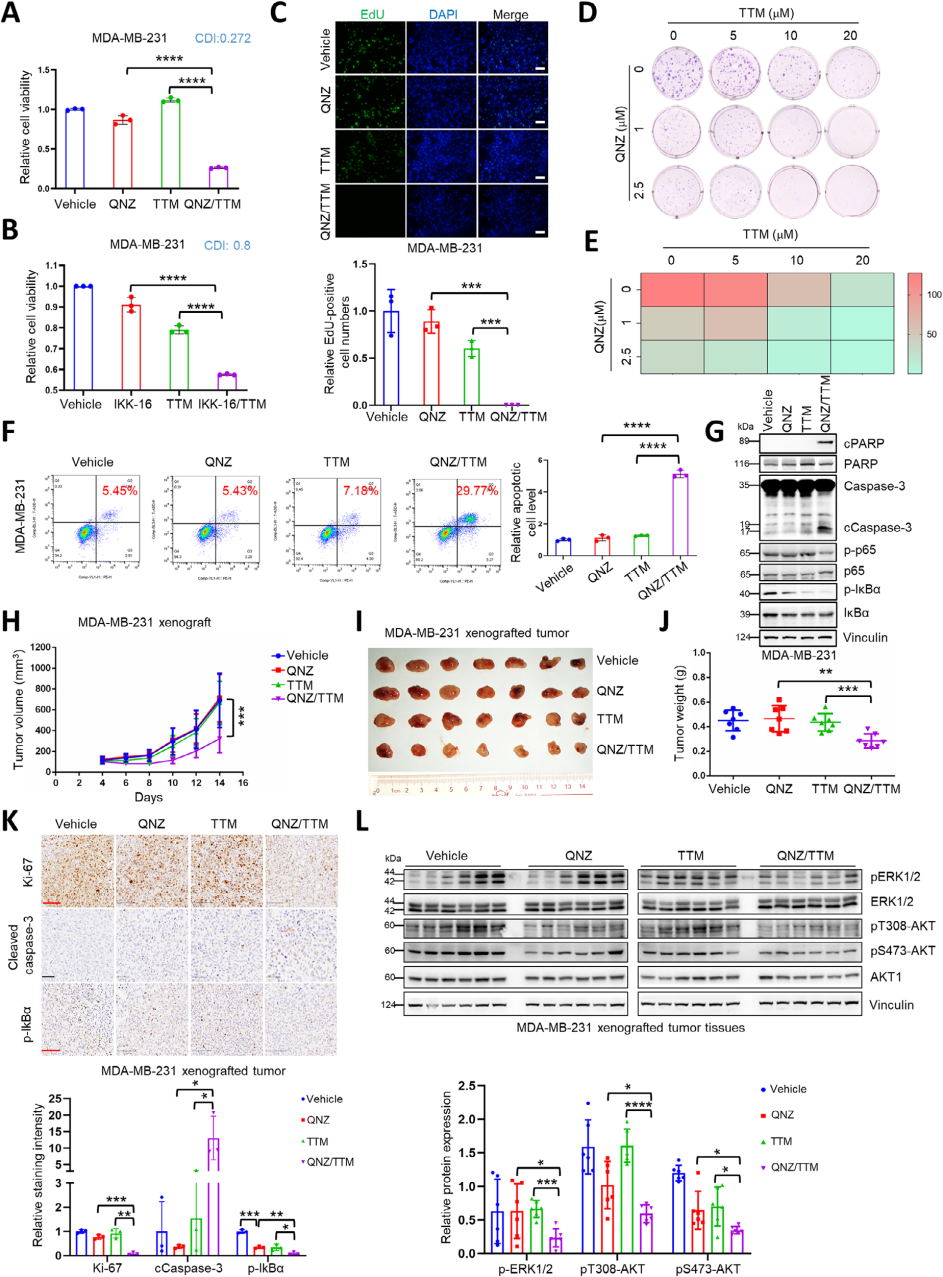
Combination of NF‐κB inhibitor and copper chelator for cancer intervention in breast cancer cells and in vivo. A,B) MDA‐MB‐231 cells were treated with indicated concentrations of QNZ (A, 8 µm) or IKK‐16 (B, 0.5 µm) and TTM (A, 30 µm; B, 40 µm) individually or in combination for 24 h, then the cell viabilities were detected and analyzed using student *t‐*test (mean+/‐SD, n = 3) (****p < 0.0001). Coefficient of drug in interaction (CDI) was calculated. CDI<1 means synergistic effect, CDI<0.7 means significantly synergistic effect. (C) MDA‐MB‐231 cells were treated with QNZ (10 µm) and TTM (30 µm) individually or in combination for 12 h, and the resulting cells were fixed and labeled with EdU (top panel), relative EdU‐labeled cell numbers were normalized and plotted (bottom panel). The results were analyzed using student *t‐*test (mean+/‐SD, n = 3) (****p < 0.0001). Bar indicates 100 µm. D,E) MDA‐MB‐231 cells were treated with indicated concentrations of QNZ and TTM individually or in combination for colony formation assay. The resulting cells were fixed and stained with crystal violet solution (D) and heat map of quantified colony numbers was plotted (E). F,G) MDA‐MB‐231 cells were treated with QNZ (8 µm) and TTM (35 µm) individually or in combination for 24 h, the resulting cells were subjected to Annexin V‐PE/7‐AAD‐labeled apoptosis assays (F, left panel) and IB analysis (G). Apoptotic cells were quantified and analyzed (F, right panel) using student *t* test (mean+/‐SD, n = 3) (****p < 0.0001). H–L) MDA‐MB‐231 cells were subjected to xenograft assay. The mice bearing MDA‐MB‐231 xenografts were treated with QNZ and TTM individually or in combination. The tumor size was monitored (H) (mean+/‐SD, n = 7) (***p < 0.001, *ANOVA* test). The tumors were dissected and weighed (I‐J) (mean+/‐SD, n = 7) (**p < 0.01, ***p < 0.001, student *t‐*test). The tumors were subjected to IHC assay (K, top panel) and IB analysis (L, top panel) with indicated antibodies. Red bar indicates 100 µm. Black bar indicates 50 µm. The IHC staining intensity (K, bottom panel) and relative protein expression (L, bottom panel) were measured and plotted. The results were analyzed using student *t‐*test (mean+/‐SD, n = 3) (*p < 0.05, **p < 0.01, ***p < 0.001).

Although accumulating inhibitors targeting the NF‐κB pathway have been approved for inflammatory diseases, and explored for cancer therapy in clinical trials,^[^ ^33^ ^]^ the cell toxicity and immune‐related side effects limit their usage in combating cancer.^[^ ^34^, ^35^ ^]^ One possible reason for this limitation is the elevation of CTR1 and copper uptake, leading to activation of MAPK and AKT pathways, resulting in resistance to NF‐κB inhibitors such as QNZ. Thus, we inferred that copper‐deprived cells with copper chelator TTM were more sensitive to QNZ. To address this, we combined the NF‐kB inhibitor with the copper chelator in different breast cancer cells (Figure S8, Supporting Information), and observed that this combined therapy strongly enhanced treatment efficacy compared to individual treatments by decreasing cell growth (**Figure** **5**A–C; Figures S9A–C and S10A–D, Supporting Information), colony formation (Figure 5D,E; Figure S9D,E, Supporting Information), and increasing cellular apoptosis (Figure 5F,G; Figures S9F–H and S10E–G, Supporting Information). Moreover, the combination of TTM and QNZ synergized to repress human breast tumor growth and promote tumor apoptosis in xenograft mouse models without markedly affecting mouse body weight (Figure 5H–K; Figure S11A, Supporting Information), accompanied by decreased MAPK and AKT activities (Figure 5L), as well as, repress mouse breast tumor growth without markedly affecting mouse body weight (Figure S11B–F, Supporting Information), accompanied with reduced PD‐L1 expression and increased CD8^+^ cells (Figure S11G, Supporting Information).

**Figure 6:**
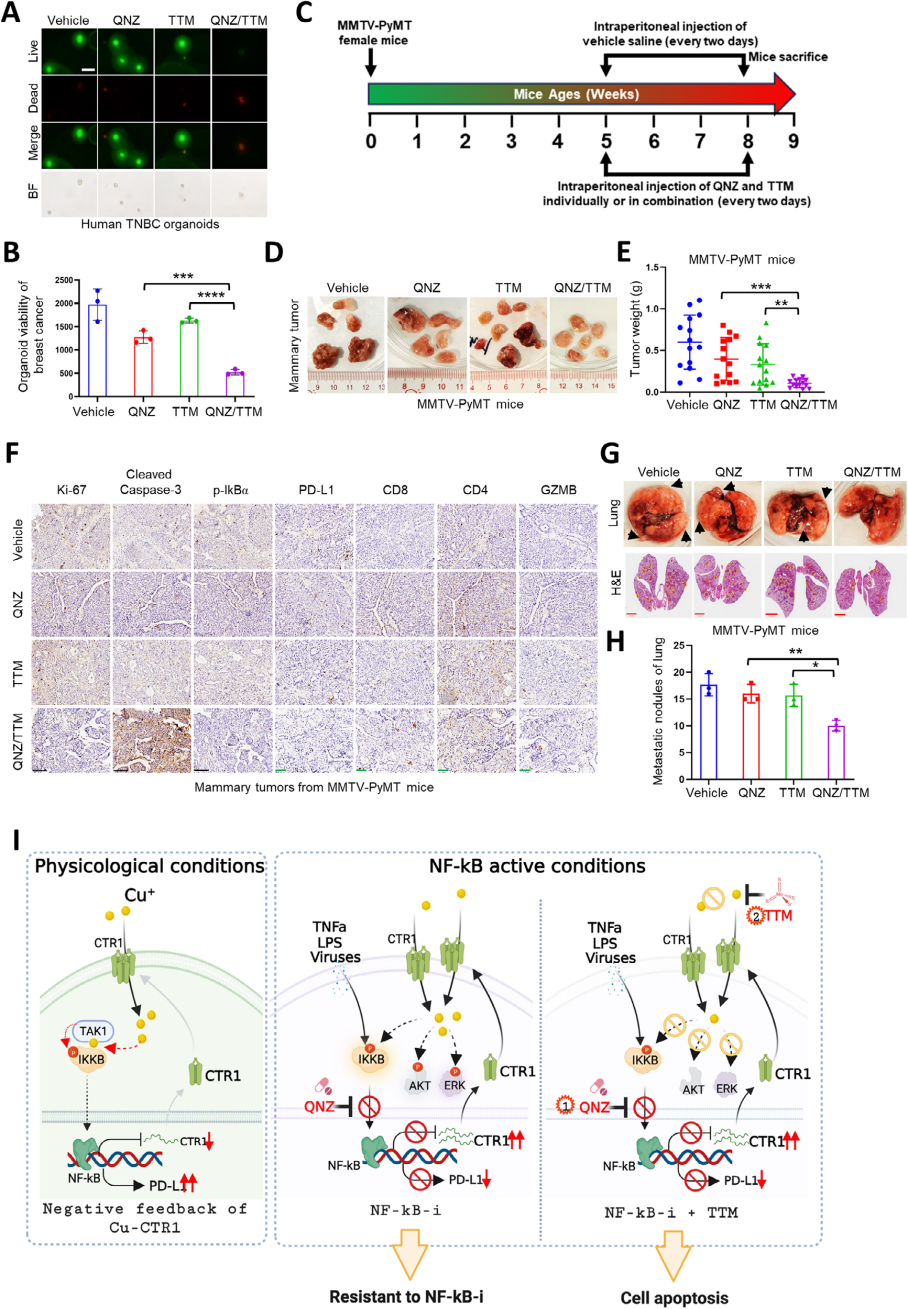
NF‐κB inhibitor synergizes with copper chelator for treating breast cancer organoids and in MMTV‐PyMT mice. A,B) Breast cancer organoids were treated with QNZ (5 µm) and TTM (20 µm) individually or in combination for 96 h, then the live and dead organoids were stained (A), the viabilities of organoids were detected and analyzed (B) using student *t*‐test (mean+/‐SD, n = 3) (***p < 0.001, ****p < 0.0001). Bar indicates 50 µm. C) The time schematic of female MMTV‐PyMT mice treated with QNZ and TTM individually or in combination. D–H) Female MMTV‐PyMT mice were treated with indicated concentrations of QNZ and TTM individually or in combination for indicated time point. The mice were euthanized, and then the tumors and lungs were dissected (D and G). The tumor weights (E) (n = 6) and lung lesions (G‐H) (n = 3) of all mice were recorded and analyzed using student *t‐*test (mean+/‐SD) (*p < 0.05, **p < 0.01, ****p < 0.0001). The mammary tumors of mice were subjected to IHC staining assay (F) with indicated antibodies. The whole lungs of all mice were sliced and disposed with H&E staining (G). Red bar indicates 2 mm. Black bar indicates 100 µm. Green bar indicates 50 µm. I) A proposed model for the potential roles of copper‐NF‐κB‐CTR1 axis in breast cancer. Under physiological conditions, copper activates NF‐κB pathway to increase PD‐L1 expression via facilitating the binding of activated TAK1 and IKKβ and further promoting IKKβ phosphorylation, whereas activated NF‐κB signaling suppresses CTR1 expression (left panel). Under NF‐κB active conditions, NF‐κB inhibitor QNZ restrains NF‐κB activation stimulated by cytokines (TNFα), LPS or viruses to reduce PD‐L1 expression, but increase CTR1 expression, further facilitating copper uptake and activating oncogenic AKT and ERK pathways, which possibly antagonizes to QNZ tumor suppressor roles (middle panel). Thus, this finding provides a strategy to combine NF‐κB inhibitor QNZ with copper chelator TTM for breast cancer therapy (right panel). The figure was generated with Biorender.com.

To further explore the potential application of combination of NF‐κB inhibitors with copper chelators, we employed human breast cancer organoids, and observed that the combination therapy dramatically promoted organoid destruction and apoptosis both in HER2‐positive and triple negative breast cancer derived organoids (**Figure** **6**A,B; Figure S12A–D, Supporting Information). Additionally, in MMTV‐PyMT mammary cancer mouse model, the combination of QNZ with TTM efficiently diminished mammary tumor growth and lung metastasis with mild effect on mouse body weight and toxicity to liver or kidney tissues (Figure 6C–H; Figure S13A–E, Supporting Information), accompanied by decreased NF‐κB activity and MAPK/AKT activity (Figure 6F; Figure S13F,G, Supporting Information). Moreover, the combination of QNZ and TTM synergized to modulate the immune microenvironment by decreasing PD‐L1 expression and increasing CD8^+^ and CD4^+^ T cells and the secretion of Granzyme B (GZMB) (Figure 6F). These findings suggest that targeting the copper‐CTR1 axis could offer a novel strategy to synergize NF‐κB inhibitors for breast cancer therapy (Figure 6I).

### 2.8 Combination of NF‐κB Inhibitor with Cuproptosis Inducer for Breast Cancer Treatment

**Figure 7:**
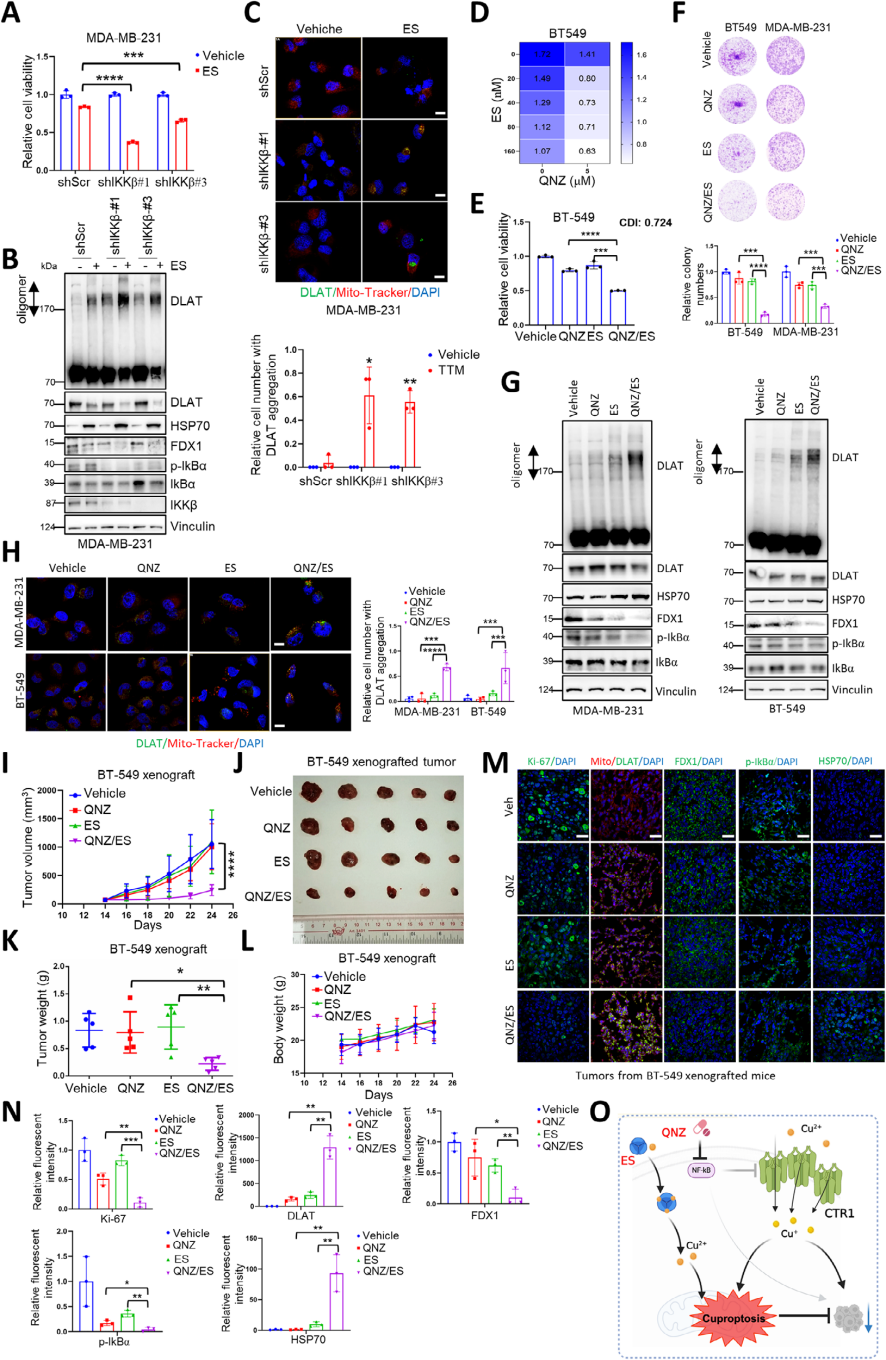
NF‐κB inhibitor synergizes with cuproptosis inducer for treating breast cancer. A) *IKKβ* knock‐down MDA‐MB‐231 and control cells were disposed with elesclomol (ES, 3.2 µm) for 48 h. The cell viabilities were detected and analyzed using student *t* test (mean+/‐SD, n = 3) (***p < 0.001, ****p < 0.0001). B) *IKKβ* knock‐down MDA‐MB‐231 and control cells were treated with elesclomol (ES, 250 nm) for 48 h. The resulting cells were harvested and then subjected to IB analysis. C) *IKKβ* knock‐down MDA‐MB‐231 and control cells were disposed with elesclomol (ES, 500 nm) for 48 h. After adding the mitochondrial dye PK Mito Deep Red (250 nm) for 2 h. The resulting cells were fixed, permeated and stained (top panel). Bar indicates 10 µm. The cell numbers with DLAT aggregation were counted and plotted (bottom panel). The results were analyzed using student *t‐*test (mean+/‐SD, n = 3) (*p < 0.05, **p < 0.01). D) BT‐549 cells were treated with different concentrations of QNZ and elesclomol (ES) for 48 h. The heatmap was plotted and indicated the synergistic effect of QNZ and ES. E) BT‐549 cells were treated with QNZ (10 µm) and ES (20 nM) for 48 h. The cell viabilities were detected and analyzed using student *t‐*test (mean+/‐SD, n = 3) (***p < 0.001, ****p < 0.0001). Coefficient of drug in interaction (CDI) was calculated. CDI<1 means synergistic effect, CDI<0.7 means significantly synergistic effect. F) BT‐549 cells were treated with QNZ (1 µm) and ES (5 nm) for colony formation assay. The resulting cells were fixed and stained with crystal violet solution (top panel) and the colony numbers were counted and plotted (bottom panel). The results were analyzed using student *t‐*test (mean+/‐SD, n = 3) (***p < 0.001, ****p < 0.0001). G) MDA‐MB‐231 (left panel) and BT‐549 (right panel) cells were disposed with QNZ (5 µM) and ES (20 nM) for 48 h, respectively. The resulting cells were collected and then subjected to IB analysis. H) MDA‐MB‐231 and BT‐549 cells were disposed with QNZ (5 µm) and ES (MDA‐MB‐231, 20 nm; BT‐549, 80 nm) for 48 h, respectively. After adding the mitochondrial dye PK Mito Deep Red (250 nm) for 2 h. The resulting cells were fixed, permeated and stained (left panel). Bar indicates 10 µm. The cell numbers with DLAT aggregation were counted and plotted (right panel). The results were analyzed using student *t‐*test (mean+/‐SD, n = 3) (***p < 0.001, ****p < 0.0001). I–N) BT‐549 cells were subjected to xenograft assay. The mice bearing BT‐549 xenografts were treated with QNZ and ES individually or in combination. The tumor size (I) and body weight of mouse (L) were monitored (mean+/‐SD, n = 5) (****p < 0.0001, *ANOVA* test). The tumors were dissected and weighed (J‐K) (mean+/‐SD, n = 5) (*p < 0.05, **p < 0.01, student *t‐*test). The tumors were subjected to IF assay (M) with indicated antibodies. The mitochondria were labelled using anti‐TOM20 antibody. Bar indicates 50 µm. The fluorescent intensity was measured and plotted (N) (mean+/‐SD, n = 3) (*p < 0.05, **p < 0.01, ***p < 0.001, student *t* test). O) NF‐κB inhibitor QNZ represses NF‐κB activation to increase CTR1 expression, further facilitating copper uptake. Meanwhile, cuproptosis inducer elesclomol (ES) delivers copper to mitochondria to cause cuproptosis. Combination of QNZ and ES further intensifies cuproptosis to inhibit tumor growth.

Due to the potent role of CTR1 in mediating elesclomol (ES)‐induced cuproptosis and the negative regulation of CTR1 by NF‐κB activation, we sought to exploit the potential strategy of NF‐κB inhibitor with ES. To this end, we depleted *IKKβ*, which could block NF‐κB pathway, and observed that blocking NF‐kB could sensitize cancer cell to ES treatment (**Figure** **7**A), accompanied by increased oligomer of DLAT and increased HSP70 levels (Figure 7B; Figure S14A, Supporting Information), as well as the aggregation of DLAT in the mitochondria (Figure 7C), hallmarks of cuproptosis.^[^ ^17^ ^]^ Furthermore, we employed the NF‐κB inhibitor QNZ for the function in combined with ES, which could markedly repress cell viability and colony formation (Figure 7D–F; Figure S14B–E, Supporting Information), coupled with increases of cuproptosis markers (Figure 7G,H; Figure S14F,G, Supporting Information). As such, the in vivo studies of combination of QNZ with ES has been performed, which strongly decreased tumor growth and enhanced cuproptosis compared with individual treatment (Figure 7I–N; Figure S14H–M, Supporting Information), with a tolerance duration. These findings provide an alternative strategy to promote cuproptosis for breast cancer therapies (Figure 7O).

## 3 Discussion

Accumulating evidence has shown that the trace metal elements such as manganese (Mn), copper (Cu), and iron (Fe) play unexpected roles in immune regulation and tumorigenesis.^[^ ^20^, ^36^, ^37^ ^]^ Among these, copper has been demonstrated by our group and others to play oncogenic roles, particularly by directly binding to MEK, PDK1, and ULK to activate downstream signaling pathways such as ERK, AKT, and autophagy, thereby promoting tumor growth in melanoma, breast cancer and NSCLC.^[^ ^14^, ^15^, ^16^ ^]^ However, the influence of copper on the tumor microenvironment (TME) has not been well explored. In line with previous reports,^[^ ^27^ ^]^ we observed that the copper‐CTR1 axis plays an essential role in the physiological or pathological activation of the NF‐κB pathway by directly binding to TAK1 to promote its K63‐linked ubiquitination, activate IKKβ and enhance p65 nuclear translocation. Consequently, downstream cytokines such as TNFα, IL‐6 and IL‐1β and the immune checkpoint PD‐L1 could be remodeled upon copper accumulation. This could attribute to the secretion of cytokines or chemokines that remodel the TME to affect tumorigenesis. However, whether other downstream targets of TAK1, including p38, JNK, involve in copper functions, meanwhile, the crosstalk between AKT/ERK and NF‐κB pathways upon copper accumulation, may provide a more complicated oncogenic mechanism for the copper‐CTR1 axis, which warrants further investigation.

**Figure 8:**
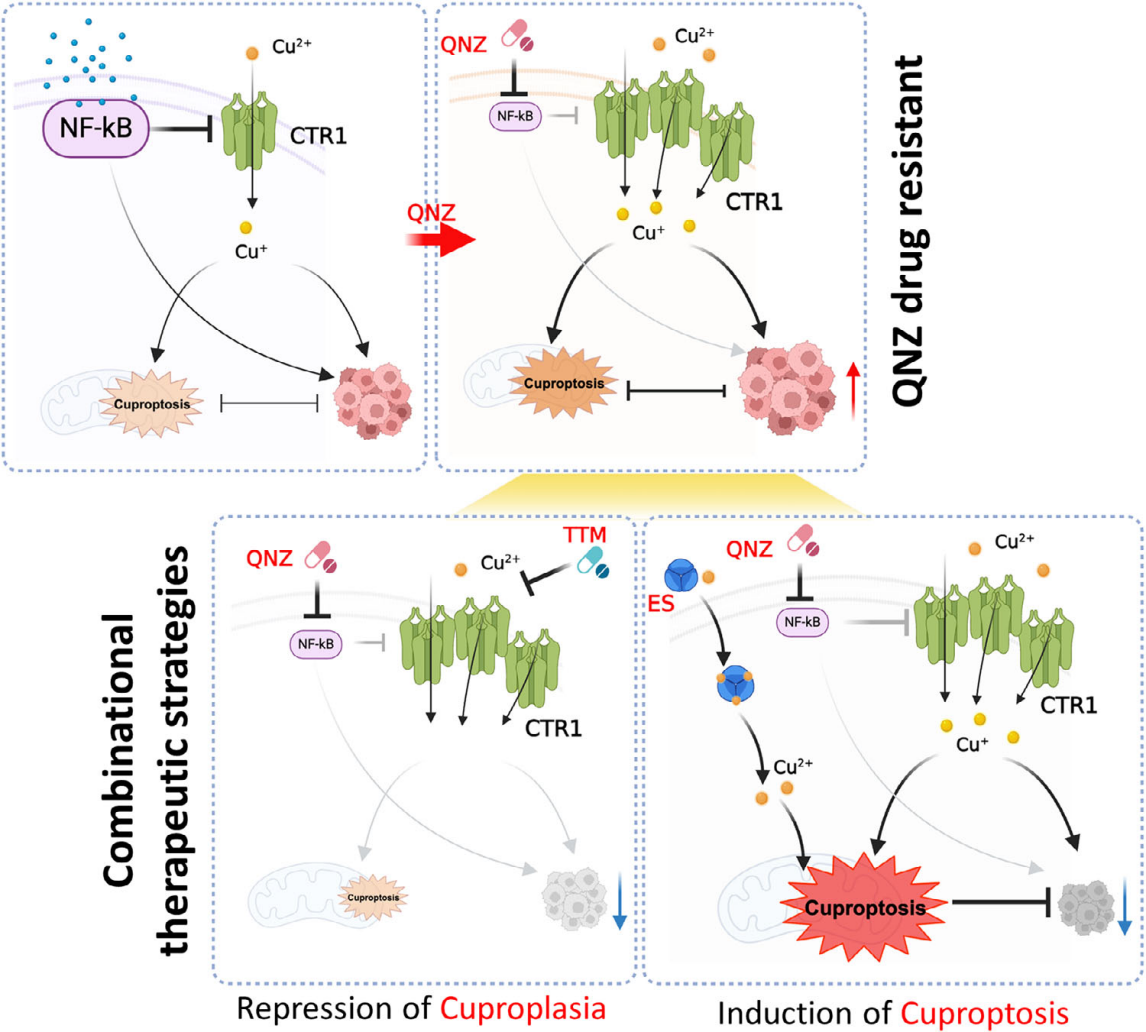
Model of targeting NF‐κB with copper chelator or cuproptosis inducer for cancer therapy. Copper activates NF‐κB signaling to accelerate tumor progression, and yet activated NF‐κB suppresses CTR1 expression to restrain copper uptake, whereas, NF‐κB inhibitor QNZ increases CTR1 expression to promote copper uptake to activate oncogenic AKT and ERK pathways, which antagonizes to QNZ tumor suppressor roles (top panel). Thus, while NF‐κB inhibitor QNZ have no good outcome in cancer therapy, combination of copper chelator TTM or cuproptosis inducer elesclomol (ES) provides a novel strategy for overcoming QNZ drug resistance in cancer therapy (bottom panel). The figure was generated with Biorender.com.

Apart from acting as an oncogenic axis, excessive accumulation of copper could also induce cell death via a TCA cycle‐specific death manner, termed as cuproptosis.^[^ ^17^ ^]^ Thus, the amount or balance of cellular copper will determine cell fate. Investigating the copper‐CTR1 negative feedback loop will provide a novel clue for this regulation. Although previous work has indicated the potential negative regulation of copper on CTR1 levels without a clear mechanism,^[^ ^38^ ^]^ here we demonstrated that NF‐κB could negatively regulate *CTR1* expression transcriptionally under both physiological and pathological conditions. Moreover, the direct binding of p65 to the promoter region of *CTR1* has been defined and validated. Given the potential role of copper‐CTR1 in positively activating the NF‐κB pathway, we show a negative regulation of NF‐κB to CTR1, thus forming a negative feedback loop on copper regulation of CTR1 to maintain copper uptake balance (**Figure** **8**). Additionally, the copper‐CTR1 axis provides a negative feedback loop on the NF‐κB pathway to maintain NF‐κB balance. Therefore, signaling that disrupts the copper‐NF‐κB balance will abnormally affect these two pathways, leading to abnormal inflammation and tumorigenesis. Hence, alternations in the TME or genomes that could disturb copper‐NF‐κB balance need to be further explored to provide new evidence for inflammation‐related diseases.

Targeted therapies have been developed and widely applied in the treatment of breast cancer.^[^ ^39^, ^40^, ^41^, ^42^ ^]^ As a dominant determinant of chronic inflammation and tumorigenesis, the TNFα‐NF‐κB pathway has also long been considered a potential target for therapies of these disease.^[^ ^33^ ^]^ However, due to side‐effects such as suppressing the immune system and leading to infection, or less response or acquired resistance for cancer therapy, the utilization of NF‐κB inhibitors has been limited, despite significant efforts.^[^ ^34^, ^35^ ^]^ In this study, we show that copper is essential for NF‐κB pathway activation, allowing us to block copper with its chelator to partially repress NF‐κB signaling and diminish inflammation and tumorigenesis (Figure 8). Additionally, in NF‐κB‐activated cancers, in particular breast cancer, inhibition of the NF‐κB pathway with its inhibitors could promote CTR1‐copper upregulation, thus elevating MEK‐ERK and PDK1‐AKT pathways to form acquired resistance to NF‐κB inhibitors. In this scenario, the combination of a copper chelator will deplete copper and, in turn, suppress ERK and AKT pathways to sensitize NF‐κB inhibitors, as validated in different subtypes of breast cancer cells, xenografted tumors, diverse of subtypes of patients’ organoids and MMTV‐PyMT mouse mammary cancer. On the other hand, instead of chelating copper, with CTR1 elevation, the combination of copper ionophores such as ES has been validated to promote the cuproptosis. Under this condition, less doses of NF‐κB inhibitor will synergize with ES to kill breast cancer, as an alternative option for cancer therapies (Figure 8). Although two approaches either repressing cuproplasia or inducing cuproptosis have been proposed and validated here to efficiently combat breast cancer, the choice of which for potential clinical application should be further validated by employing different subtypes of patient derived xenografts (PDX) mouse models or humanized mouse models. Meanwhile, although we have utilized lower doses of NF‐κB inhibitors to mitigate side effects, some cytotoxicity was also present in immunocompetent mice with tolerance. Therefore, optimizing therapeutic windows for NF‐κB inhibitors and copper chelators/cuproptosis inducers to achieve greater efficacy and fewer side effects will provide a promising strategy for chronic inflammation‐induced cancers (Figure 8).

## 4 Experimental Section

### Cell Culture

Breast cancer cell lines including MCF‐7, T‐47D, BT‐474, ZR‐75‐30, SKBR‐3, BT‐549, MDA‐MB‐453, MDA‐MB‐231, MDA‐MB‐468, 4T1, and E0771 were cultured in RPMI 1640 medium or DMEM medium supplemented with 10% fetal bovine serum (FBS), penicillin (100 units) and streptomycin (100 µg mL^−1^), and kept in our lab. MEFs *Ctr1^‐/‐^* and MEFs *Ctr1^+/+^* were used as previously.^[^ ^26^ ^]^ HEK293T cells were cultured in DMEM medium supplemented with 10% FBS. Cell transfection was performed using Lipofectamine 2000 (Invitrogen) or PEI (Polysciences) according to the manufacturer's instructions. Packaging of knock‐down or over‐expression viruses, as well as subsequent infection of various cell lines were performed according to the methods described previously.^[^ ^16^, ^26^ ^]^ After viral infection, cells were maintained with different concentrations of hygromycin or puromycin, depending on cell lines and the viral vectors used to infect cells.

### Plasmids

The construct pLKO.1‐shCTR1‐tet‐on,^[^ ^16^, ^26^ ^]^ NF‐κB luciferase reporter plasmid,^[^ ^43^ ^]^ and the dominant‐negative plasmids of IκBα (IκBα‐DN)^[^ ^44^ ^]^ were previously described. The fragments HA‐TAK1, HA‐TAK1‐N (300 amino acids fragment from N‐terminal), HA‐TAK1‐C (306 amino acids fragment from C‐terminal), HA‐TAK1‐H154A, HA‐TAK1‐M196A, HA‐TAK1‐2A, Flag‐TRAF2, Flag‐TRAF6, His‐Ub‐K63O, Myc‐TAK1, Flag‐IKKβ, HA‐MEK, and HA‐ERK1 were cloned and inserted into the vectors pcDNA3.1 and pLenti‐Hygro, respectively. The fragment IKKβ was cloned and inserted into the vector pCMV‐GST. The shRNAs of IKKβ or TAK1 were inserted into the vector pLKO.1, respectively. The fragment of *SLC31A1* promoter was cloned and inserted into the vector pGL3‐basic. The 300 amino acids fragment from N‐terminal of TAK1 (TAK1‐N) and the mutant (TAK1‐N‐2A) and 20 amino acids fragment of IκBα were cloned into the vector pGEX‐4T1. Details of plasmid constructions are available upon request. All plasmids were generated using the QuikChange XL Site‐Directed Mutagenesis Kit (Stratagene) according to the manufacturer's instruction.

### Reagents and Antibodies

CuSO_4_ (C805782) and NH_4_Cl (A801304) were purchased from Macklin. Doxycycline (DOX) (D1515), TTM (323446), and LPS (L2630) were purchased from Sigma. Recombinant Human TNFα (300‐01A) and Recombinant Murine TNFα (315‐01A) were obtained from PEPROTECH. Human TAK1 Protein (HY‐P703591) was purchased from MCE. NF‐κB inhibitor QNZ (S4902), NF‐κB inhibitor JSH‐23 (S7351), NF‐κB inhibitor PDTC (S3633), TAK1 inhibitor Takinib (S8663), IKKβ inhibitor IKK‐16 (S2882), Elesclomol (S1052), MG132 (S2619), 3‐MA (S2767), HCQ (S4430), MK‐2206 (S1078), and Trametinib (S2673) were obtained from Selleck. All antibodies were used at a 1:2000 dilution in TBST buffer with 5% bovine serum albumin (BSA) for western blot. Anti‐phospho‐Ser176/180‐IKKα/β antibody (2697), anti‐phospho‐Ser32‐IκBα antibody (2859), anti‐IκBα antibody (4812), anti‐phospho‐Ser536‐p65 antibody (3033), anti‐p65 antibody (8242), anti‐phospho‐ERK1/2 antibody (4370), anti‐ERK1/2 antibody (4695), anti‐PD‐L1 antibody (13684), anti‐PD‐L2 antibody (82723), Anti‐CTR1 antibody (13086), anti‐phospho‐Thr308‐AKT antibody (2965), anti‐phospho‐Ser473‐AKT antibody (4060), anti‐AKT1 antibody (2938), anti‐Caspase‐3 antibody (14220), anti‐PARP‐antibody (9532), anti‐Cleaved PARP antibody (5625), anti‐rabbit IgG (H+L), F(ab“)_2_ Fragment (Alexa Fluor 488 Conjugate) (4412), and anti‐mouse IgG (H+L), F(ab”)_2_ Fragment (Alexa Fluor 594 Conjugate) (8890) were obtained from Cell Signaling Technology. Anti‐PD‐L1 antibody (EPR20529) and anti‐CD8 antibody (ERP21769), and anti‐GZMB antibody (ab255598) were obtained from Abcam. Anti‐CTR1 polyclonal antibody (NB100‐402) was obtained from Novus Biologicals. Anti‐IKKβ antibody (15649‐1‐AP), anti‐TAB1 antibody (67020‐1‐Ig), anti‐TAB2 antibody (14410‐1‐AP), anti‐p65 antibody (10745‐1‐AP), anti‐TAK1 antibody (67707‐1‐Ig), anti‐CCS antibody (22802‐1‐AP), anti‐DLAT antibody (13426‐1‐AP), anti‐FDX1 antibody (12592‐1‐AP), anti‐TOM20 antibody (66777‐1‐Ig), anti‐P62/SQSTM1 antibody (18420‐1‐AP), anti‐PD‐L1 antibody (66248‐1‐Ig), anti‐pan‐keratin antibody (26411‐1‐AP), anti‐CD4 antibody (67786‐1‐Ig), and anti‐GST‐tag antibody (66001‐1‐Ig) were obtained from Proteintech. Anti‐Vinculin antibody (V4505), monoclonal anti‐Flag antibody (F‐3165, clone M2), and agarose conjugated‐anti‐HA antibody (A2095) were obtained from Sigma. Anti‐Actin antibody (sc‐69879), protein A/G plus‐agarose (sc‐2003) and anti‐HSP70 antibody (sc‐24) were obtained from Santa Cruz. Monoclonal anti‐HA antibody (901503) and APC anti‐human CD274 (B7‐H1, PD‐L1) antibody (329708) were obtained from BioLegend. Anti‐TAK1 antibody (A19077), anti‐XIAP antibody (A20846) and anti‐phospho‐Ser32‐IκBα antibody (AP0731) were purchased from ABclonal. Glutathione Sepharose 4B (17‐0756‐05) was obtained from GE Healthcare. NTA agarose (30310) and Ni‐NTA agarose (30230) were purchased from QIAGEN. Peroxidase‐conjugated anti‐mouse secondary antibody (115‐035‐003) and peroxidase‐conjugated anti‐rabbit secondary antibody (111‐035‐003) were obtained from Jackson ImmunoResearch. Peroxidase‐conjugated mouse anti‐rabbit IgG LCS (A25022, 1:10000) was obtained from Abbkine. Anti‐Phospho‐Ser10‐Histone H3 antibody (ET1601‐30) were obtained from HUABIO (Zhejiang, China).

### Dual‐Luciferase Reporter Assay

NF‐κB luciferase reporter plasmid and the reporter plasmids containing p65‐*SLC31A1* promoter binding region were applied in dual‐luciferase reporter assays. The cells transfected with different reporter plasmids were disposed with diverse experiment conditions. The assay was performed following the manufacturer's instructions of Dual‐Luciferase Reporter Assay System (E1960, Promega).

### Immunoblotting (IB), Immunoprecipitation (IP) Analyses and Immunofluorescence (IF)

The IB and IP assays were carried out according to the previously described.^[^ ^16^, ^26^ ^]^ The immunofluorescent assays were performed and analyzed according to the previously described.^[^ ^26^ ^]^ The cells were incubated with the primary antibodies of anti‐p65 antibody (1:200), anti‐PD‐L1 antibody (1:200) or anti‐CTR1 antibody (1:200), respectively. The tumor tissues were incubated with the primary antibodies of anti‐Ki‐67 antibody (1:200), anti‐DLAT antibody (1:200), anti‐FDX1 antibody (1:200), anti‐phospho‐IκBα antibody (1:200) or anti‐HSP70 antibody (1:200), respectively.

### Detection of DLAT Oligomers and Aggregation

For DLAT oligomers, the cells were treated with elesclomol (ES) individually or in combination with QNZ for indicated time points. Following, the cells were collected and lysed in EBC buffer (50 mm Tris PH7.5, 120 mm NaCl, 0.5% NP‐40) containing EDTA‐free protease inhibitor and phosphatase inhibitor. The lysates were added with Dithiothreitol‐free SDS‐PAGE sample loading buffer and then heated at 70 °C for 10 min. The resulting samples were subjected to 6% SDS‐PAGE. For DLAT aggregation, the cells were disposed with ES individually or in combination with QNZ for indicated time points. Then the cells were treated with 250 mM mitochondrial dye PK Mito Deep Red (Genvivo, China, PKMDR‐2) for 2 h. The cells were incubated with the primary antibodies of anti‐DLAT antibody (1:200) for IF analysis.

### Ubiquitination Assay

The plasmid HA‐TAK1 or HA‐TAK1‐2A and His‐Ub‐K63O were cotransfected into HEK293T cells and subsequently treated with different agents. The resulting cells were harvested and lysed in buffer A (6 m Guanidine Hydrochloride, 0.1 m Na_2_HPO_4_/NaH_2_PO_4_, 10 mm Imidazole, PH8.0) and then incubated with Ni‐NTA beads for 3 h at RT. Next, the beads were wash 4 times including 1 time using buffer A, 1time using buffer A and buffer Ti, 2 times using buffer Ti (25 mM Tris‐HCl, 20 mM Imidazole, PH6.8).

### Hematoxylin‐Eosin (H&E) and Immunohistochemical (IHC) Staining

Tissue sections were stained following the manufacturer's instructions of H&E staining kit (G1120, Solarbio, Beijing, China). The breast cancer tissues and adjacent normal breast tissues were obtained from biological specimen banks of the First Affiliated Hospital, Sun Yat‐sen University (Guangzhou, China). The IHC staining of section was carried out following the previously described.^[^ ^26^ ^]^

### In Vivo Copper Binding and Molecular Docking

Copper‐PDC beads were purchased from Affiland. In vivo copper binding was performed as described previously.^[^ ^16^ ^]^ TAK1‐H154 and TAK1‐M196 were obtained via aligning the amino acid sequence of human MEK1 and TAK1 using Clustal W. Molecular docking studies were carried out using Schrodinger software package (Schrodinger, LLC, New York, NY). The coordinates of the TAK1 were generated based on the AlphaFold predicted structure and subjected to preparation using the protein preparation wizard. The CHARMM force field method and the Momany‐Rone partial charge method were used to add hydrogen atom and charge to the system, respectively.

### Copper Content Measurement

The cells were seeded in the 6‐well plate and then treated with different agents. The resulting cells were lysed in EBC buffer (50 mM Tris PH7.5, 120 mM NaCl, 0.5% NP‐40) containing EDTA‐free protease inhibitor and phosphatase inhibitor. The lysates were used for detecting copper according to manufacturer's protocol of Copper (Cu) Colorimetric Assay Kit (Elabscience, China, E‐BC‐K300‐M).

### Isolation and Purification of T Cells and T Cell–Mediated Tumor Cell Killing Assay

Assays were conducted as described previously.^[^ ^45^ ^]^ Briefly, peripheral blood mononuclear cells (PBMCs) were isolated using Lymphoprep (07811, STEMCELL). The PBMCs were resuspended in EasySep Buffer (20144, STEMCELL) and then T cells were isolated using EasySep Human CD3 Positive Selection Kit II (17851, STEMCELL) according to the manufacturer's protocol and cultured with RPMI1640 complete medium containing 10% FBS (10100147C, Gibco). To analyze T cell–mediated tumor cell killing, T cells were reactivated with Recombinant Human IL‐2 Protein (10 ng mL^−1^) (202‐IL‐010, R&D Systems) and Dynabeads Human T‐Activator CD3/CD28 (4 × 10^5^ beads mL^−1^) (11131D, Gibco) for 72 h. The indicated cancer cells (10000 cells/well) were seeded into 6‐well plates and cocultured with activated T cells at a 10:1 ratio. T cells and cancer cell debris were washed with phosphate‐buffered saline (PBS) three times after 3 days. Until the obvious colony formation, the cells were fixed with 4% paraformaldehyde for 15 min at room temperature and then stained with crystal violet. Colony numbers were counted with Image J software.

### mRNA Extraction, qPCR and RNA Sequencing

Total RNA was extracted, and Quantitative Real‐time PCR was performed according to the previously described.^[^ ^26^ ^]^ The primers were as followed: Forward primer‐hActin: CATGTACGTTGCTATCCAGGC, Reverse primer‐hActin: CTCCTTAATGTCACGCACGAT; Forward primer‐hTNFα: GCCGCATCGCCGTCTCCTAC, Reverse primer‐hTNFα: CCTCAGCCCCCTCTGGGGTC; Forward primer‐hIL‐1β: ATGGCTTATTACAGTGGC, Reverse primer‐hIL‐1β: GTAGTGGTGGTCGGAGA; Forward primer‐hIL‐6: ACTCACCTCTTCAGAACGAATTG, Reverse primer‐hIL‐6: CCATCTTTGGAAGGTTCAGGTTG; Forward primer‐mActin: GCTGACAGGATGCAGAAGGAG, Reverse primer‐mActin: TCAAAGAAAGGGTGTAAAACGC; Forward primer‐mTNFα: CAGGCGGTGCCTATGTCTC, Reverse primer‐mTNFα: CGATCACCCCGAAGTTCAGTAG; Forward primer‐mIL‐6: TAGTCCTTCCTACCCCAATTTCC, Reverse primer‐mIL‐6: TTGGTCCTTAGCCACTCCTTC; Forward primer‐hPD‐L1: TGGCATTTGCTGAACGCATTT, Reverse primer‐hPD‐L1: TGCAGCCAGGTCTAATTGTTTT; Forward primer‐hPD‐L2: GTACATAATAGAGCATGGCAGCAATG, Reverse primer‐hPD‐L2: CCACCTTTTGCAAACTGGCTGT; Forward primer‐ hCTR1: CAACCTTCTCACCATCACCC, Reverse primer‐ hCTR1: AGTTCCACATTCTTAAAGCCAAAG.

The cell line MDA‐MB‐231 shCTR1‐tet on was constructed according to the previously described^[^ ^16^, ^26^ ^]^ and then disposed with DOX (1 µg mL^−1^) for 72 h to knock down *CTR1*. The breast cancer cells SK‐BR‐3 were cultured with serum‐free DMEM medium and disposed with CuSO_4_ (100 µM) overnight. The resulting cells were collected for extracting high‐quality total RNA for RNA‐seq. RNA‐seq libraries were prepared by BGIseq500 platform (BGI‐Shenzhen, China) and were sequenced using Illumina Hi‐Seq platform. RNA‐seq reads were mapped to NCBI human genome GRCh38.p13. The raw sequencing data were filtered with SOAPnuke. Expression level of gene was calculated by RSEM (v1.3.1). Differential expression analysis was performed using the DESeq2 (v1.4.5) with Q value ≤ 0.05. GO (http://www.geneontology.org/) and KEGG (https://www.kegg.jp/) enrichment analysis of annotated different expression gene was performed by Phyper based on Hypergeometric test. The significant levels of terms and pathways were corrected by Q value with a rigorous threshold (Q value ≤ 0.05).

### ChIP‐qPCR

ChIP assay was conducted using SimpleChIP Plus Enzymatic Chromatin IP Kit (Magnetic Beads) (9005, CST) according to the manufacturer's instructions. Anti‐p65 antibody (8242, CST) and Rabbit IgG Isotype Control (10500C, Invitrogen) were used for ChIP assay. The p65 ChIP‐Seq data, including bigwig and bed files, were downloaded from GEO database under GSE52469. *SLC31A1* gene tracks were visualized using the IGV (version 2.17.0) genome browser, along with annotation tracks. The putative binding sites of p65‐*IL‐1β* promoter were predicated using the database JASPAR at the website (https://jaspar.elixir.no/). Quantitative Real‐time PCR was performed using the SYBR Green Premix Pro Taq HS qPCR Kit II (Rox Plus) (AG11719) to verified the putative binding regions of p65‐*SLC31A1* promoter and p65‐*IL‐1β* promoter. Simultaneously, the products of ChIP‐qPCR were identified by DNA gel electrophoresis. The primers of the ChIP‐qPCR were as followed: Forward primer‐*SLC31A1* promoter: CGGGAAATCCTCGGCCTC, Reverse primer‐*SLC31A1* promoter: CCGAGAGAAGCCTTACCTTTCCA; Forward primer‐*IL‐1β* promoter: CAGAAGCAGATGCTGCCA, Reverse primer‐*IL‐1β* promoter: ATATTCGTGCAGACAGCATGT.

### shRNAs and siRNAs

Short hairpin RNAs (shRNAs) targeted the human *IKKβ* or *TAK1* transcript were inserted into the vector pLKO.1. The target sequences were as followed: shIKKβ‐#1 (5′‐CCAGCCAAGAAGAGTGAAGAA‐3′), shIKKβ‐#2 (5′‐GCTGGTTCATATCTTGAACAT‐3′), and shIKKβ‐#3 (5′‐CGGAAGTACCTGAACCAGTTT‐3′), which were the same as the target *IKKβ* sequences by small interfering RNAs (siRNAs); shTAK1‐#1 (5′‐ CGGAACCTTTAGGGATAGTTC‐3′) and shTAK1‐#2 (5′‐ CCCGTGTGAACCATCCTAATA‐3′). Small interfering RNAs targeted *p65* or *XIAP* transcript were synthesized by Suzhou Genepharma Co., Ltd. (China) and the target sequences were as followed: si‐p65‐#1 (5′‐GCCTTAATAGTAGGGTAAGTT‐3′), si‐p65‐#2 (5′‐CGGATTGAGGAGAAACGTAAA‐3′), and si‐p65‐#3 (5′‐CACCATCAACTATGATGAGTT‐3′); si‐XIAP‐#1 (5′‐AGCTGTAGATAGATGGCAATA‐3′), si‐XIAP‐#2 (5′‐CAGAATGGTCAGTACAAAGTT‐3′), and si‐XIAP‐#3 (5′‐GCACTCCAACTTCTAATCAAA‐3′).

### Purification of GST‐Tagged Proteins from Bacteria and In Vitro Kinase Assay

Recombinant GST‐IκBα fragment, recombinant GST‐TAK1‐N and GST‐TAK1‐N‐2A were prepared following the detailed procedures described previously.^[^ ^26^ ^]^ The sequences of recombinant GST‐IκBα fragment containing 20 amino acids were CGGCTACTGGACGACCGCCACGACAGCGGCCTGGACTCCATGAAAGACGAGGAGTACGAG. IκBα or IKKβ in vitro kinase assay was performed from a protocol according to the manufacturer's instructions by an AKT Kinase Assay Kit purchased from Cell Signaling Technology as previously described.^[^ ^16^, ^26^ ^]^ The kinase IKKβ or TAK1 treated with or without CuSO_4_ or/and TTM and the substrate IKKβ were immunoprecipitated and purified from HEK293T. The indicated kinases and substrates were incubated in the kinase buffer in the presence or absence of a fixed 100 µM concentration of CuSO_4_ or TTM. Recombinant GST‐TAK1‐N fragment contained 300 amino acids from N‐terminal of TAK1 and recombinant GST‐TAK1‐N‐2A fragment was mutated at H154 and M196 sites on the basis of GST‐TAK1‐N fragment. Recombinant TAK1‐N and TAK1‐N‐2A were acquired using Thrombin (Solarbio, China, T8021) to cleave the GST tag of GST‐TAK1‐N and GST‐TAK1‐N‐2A, respectively.

### Cell Proliferation, Colony Formation, and Soft Agar Assays

The detailed procedures of the assays were carried out according to the previously described.^[^ ^16^, ^26^ ^]^ For CCK8 assay, cells (1000 or 3000 cells/well) were seeded into 96‐well plates and disposed with indicated concentration of QNZ or IKK‐16 and TTM individually and in combination for the indicated time points. For colony formation assay, cells (1000 cells/well) were seeded into 6‐well plates and treated with indicated concentration of QNZ and TTM individually and in combination, following left for 8‐14 days until formation of visible colonies. Quantification of the result was performed with Image J software.

### Flow Cytometry Analysis

The indicated cancer cells were collected and stained with APC anti‐human CD274 (B7‐H1, PD‐L1) antibody for 30 mins and then were washed 3 times with PBS containing 5% FBS. For cell apoptosis, cells were treated with indicated concentration of agents individually or in combination for the indicated time points. And then, resulting cells were collected and stained using Annexin V‐PE/7‐AAD Apoptosis Detection Kit (Vazyme, A213‐01) according to the manufacturer's protocol. Stained cells above were analyzed by flow cytometry.

### EdU Staining

Assays were conducted as described previously.^[^ ^26^ ^]^ Briefly, various breast cancer cells were treated with the indicated concentration of QNZ and TTM individually and in combination for the indicated time points. The cells were disposed using Cell‐Light EdU Apollo488 In Vitro Kit (RIBOBO, C10310‐3) or Cell‐Light EdU Apollo567 In Vitro Kit (RIBOBO, C10310‐1) and analyzed by fluorescence microscope (OLYMPUS). Quantification of the fluorescent intensity was performed with Image J software.

### Mouse Models

6‐week‐old female C57BL/6J mice (Guangdong GemPharmatech Co., Ltd., China) were administered with TTM at a dose of 10 mg kg^−1^ by intraperitoneal injection every two days for a week. The mice were administered with LPS at a dose of 10 mg kg^−1^ once by intraperitoneal injection after 2 h injected with TTM at a dose of 20 mg kg^−1^. Six hours after injected with LPS, some mice were euthanized and the indicated organs were collected. Small portions of lungs and kidneys were utilized for IB analyses or fixed in formalin for H&E staining. Following, the other mice were administered with TTM at a dose of 10 mg kg^−1^ by intraperitoneal injection every day. Survival of mice were observed and recorded every day until no survival of mice individually injected with LPS. The mouse breast cancer autochthonous model MMTV‐PyMT mice (Jiangsu GemPharmatech Co., Ltd., China, female, 5‐week‐old) were individually or in combination administered with QNZ at a dose of 0.3 mg kg^−1^ or TTM at a dose of 10 mg kg^−1^ by intraperitoneal injection every other day. Until spontaneous breast tumor formation of control group, the mice were euthanized and the serum and indicated organs were collected. The serum was used for detection of drug toxicity using Alanine aminotransferase Assay Kit (C009‐2‐1, NJJCBIO, Nanjing, China) and Aspartate aminotransferase Assay Kit (C010‐2‐1, NJJCBIO, Nanjing, China) following the manufacturer's instructions. Small portions of tumors, livers, and kidneys were fixed in formalin for H&E and IHC stainings. All animal experimental procedures were approved by the Institutional Animal Care and Use Committee of Sun Yat‐sen University (Approval No.: SYSU‐IACUC‐2024‐B0900).

### Mouse Xenograft Study

Assays were conducted following the previously described.^[^ ^26^ ^]^2 x 10^6^ MDA‐MB‐231 cells or 3 x 10^6^ T‐47D cells stably expressing GFP, TAK1, 2A were respectively injected into the flank of female nude mice (GuangDong GemPharmatech Co., Ltd, 4 weeks of age). 6 x 10^5^ mouse mammary tumor cells (E0771) were injected into the flank of female C57BL/6J mice (GuangDong GemPharmatech Co., Ltd, 4 weeks of age). For mice with MDA‐MB‐231 xenografts, treatment groups were individually or in combination administered with QNZ at a dose of 1 mg kg^−1^ and TTM at a dose of 5 mg kg^−1^ by intraperitoneal injection every day. For mice with E0771 tumors, treatment groups were individually or in combination administered with QNZ at a dose of 0.4 mg kg^−1^ and TTM at a dose of 5 mg kg^−1^ by intraperitoneal injection every day. 2 x 10^6^ BT‐549 cells or 2 x 10^6^ MDA‐MB‐231 cells were respectively injected into the flank of female nude mice (GuangDong GemPharmatech Co., Ltd, 4 weeks of age). For mice with BT‐549 or MDA‐MB‐231 xenografts, treatment groups were individually or in combination administered with QNZ at a dose of 0.5 mg kg^−1^ and ES at a dose of 10 mg kg^−1^ by intraperitoneal injection every two days. Tumor size of xenografted mouse was measured every two days with a caliper. The tumor volume was determined with the formula: L x W^2^ x 0.52, where L is the longest diameter and W is the shortest diameter. Until termination of animal experiment, mice were euthanized to dissect solid tumors and then tumor weights were measured. All animal experimental procedures were approved by the Institutional Animal Care and Use Committee of Sun Yat‐sen University (Approval No.: SYSU‐IACUC‐2024‐B0900).

### Establishment of Patient‐Derived Breast Cancer Organoids

The detailed procedures of the assay were conducted using Breast Cancer Organoid Kit (K2147‐BC, bioGenous, Suzhou, China). Briefly, human breast cancer tissue pieces were collected in ice‐cold Primary Tissue Storage Solution (K601005) from the First Affiliated Hospital of Sun Yat‐sen University in compliance with the medical ethical standards and procedures. After rinsing and mincing the tissue, the tissue fragments were digested with Tumor Tissue Digestion Solution (K601003) at 37 °C. Until the digestion process was finished, the cells were filtered and collected, and then were removed the red blood cells using Red Blood Cell Lysis Solution (E238010) and washed twice using Breast Cancer Organoid Basal Medium (K2147‐BC‐A100). The resulting cells were resuspended in Organoid Culture ECM (M315066) and cultivated with breast cancer organoid complete medium containing Breast Cancer Organoid Supplement B (K2147‐BC‐B100) and Breast Cancer Organoid Supplement C (K2147‐BC‐C100). The medium was changed every 3‐4 days until the formation of patient‐derived breast cancer organoids.

### Detection of Breast Cancer Organoid Viability

Patient‐derived breast cancer organoids were passaged according to the manufacturer's protocol of Breast Cancer Organoid Kit (K2147‐BC, bioGenous, Suzhou, China). The organoids were seeded into 96‐well plates and treated with the indicated concentration of QNZ and TTM individually and in combination for the indicated time points. Then, the organoids were disposed using Live & Dead Viability/Cytotoxicity Assay Kit for Animal Cells (L6037L, UElandy, Suzhou, China) or CellTiter‐Glo Luminescent Cell Viability Assay (G7571, Promega) following the manufacturer's instructions, respectively. The disposed organoids were analyzed by fluorescence microscope (OLYMPUS) or Varioskan LUX Multimode Microplate Reader (Thermo Scientific).

### Quantification and Statistical Analyses

GraphPad Prism version 8.0 and SPSS statistic 19.0 were used for statistical analyses. For all experiments, data were analyzed by two‐tailed Student's *t*‐test or two‐way analysis of variance (*ANOVA*) test. N represents repeats of experiment. Results were considered significant at *p < 0.05, **p < 0.01, ***p < 0.001, and ****p < 0.0001.

## Conflict of Interest

The authors declare no conflict of interest.

## Author Contributions

X.Z. and Y.S. contributed equally to this work. J.G. and X.Z. performed conception and design; X.Z. and Y.S. performed Development of methodology; X.Z., Y.S., W.Y., Z.S., Z.W., B.G., J.C., Y.L., and J.L. performed acquisition of data (provided animals, acquired and managed patients, provided facilities, etc.); X.Z. and Y.S. performed analysis and interpretation of data (e.g., statistical analysis, biostatistics, computational analysis); J.G. and X.Z. wrote the manuscript; X.Z., Y.S., L.W., W.X., Z.S., X.W., Q.J., L.B., and J.L. performed administrative, technical, or material support (i.e., reporting or organizing data, constructing databases); J.G., J.L., and W.X. performed study supervision. All authors approved the manuscript.

## Supporting information



Supporting Information

## Data Availability

Data and materials are available for request.
