# Supplementary material for: Disruption of NF‐κB‐Mediated Copper Homeostasis Sensitizes Breast Cancer to Cuproptosis
Source: Adv Sci (Weinh). 2025 Sep 26;12(47):e06201. doi: 10.1002/advs.202506201 (PMC12713080; doi:10.1002/advs.202506201)
Supplement: Supplementary file 1 — Supporting Information [file ADVS-12-e06201-s001.pdf]

Supporting information for

Disruption of NF-κB-Mediated Copper Homeostasis Sensitizes Breast Cancer to Cuproptosis

Xiaomei Zhang, Yaqing Su, Weixiong Yang, Zimin Song, Zicheng Sun, Xueji Wu, Jianwen Chen, Bing Gao, Zekang Wang, Lei Wang, Qiwei Jiang, Lang Bu, Jingting Li, Ying Lin, Wei Xie, Jie Li, Jianping Guo

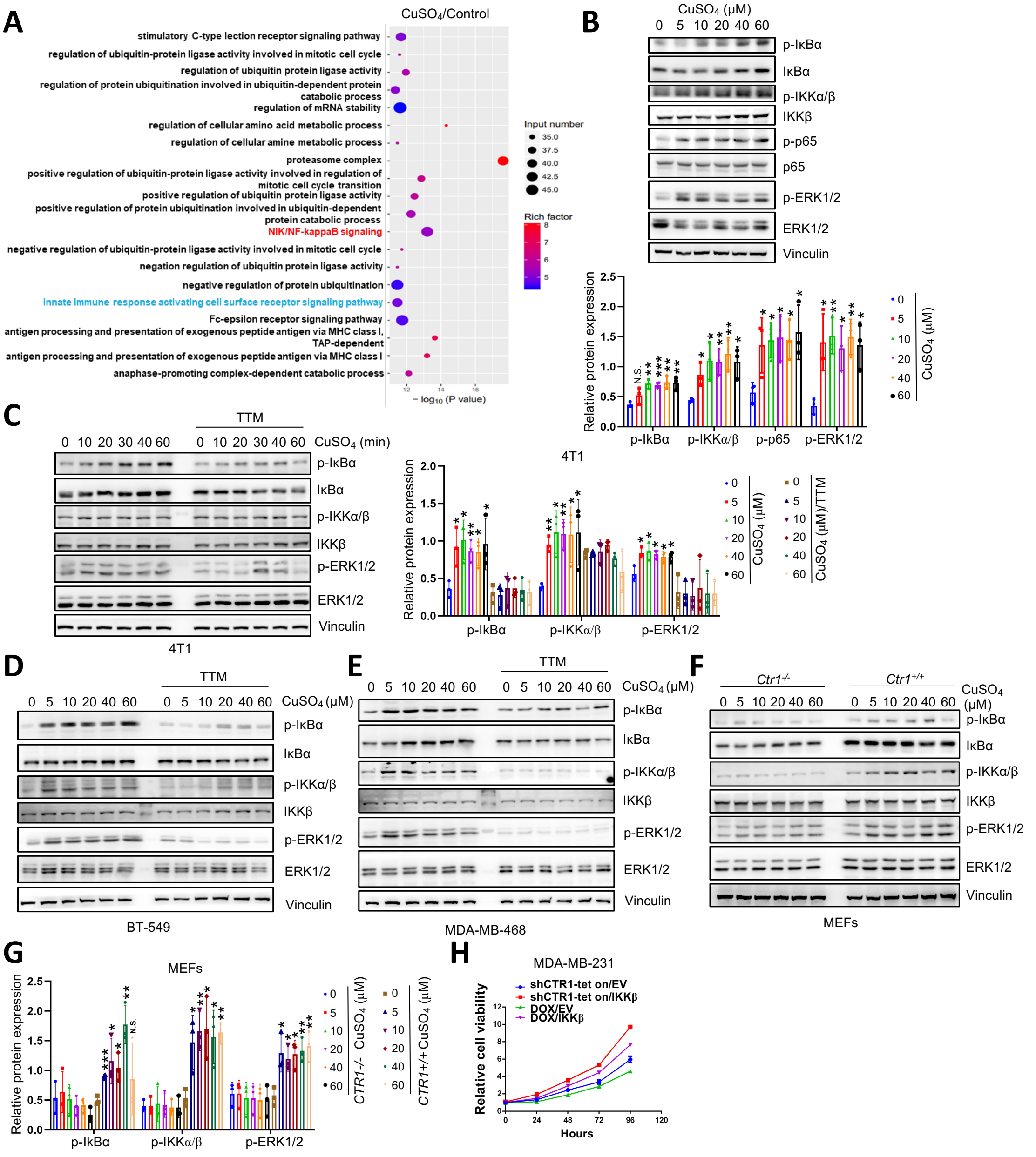

Figure S1. Copper mediates NF-κB activation and PD-L1 expression

(A) SK-BR-3 cells were disposed with $CuSO_4$ (100 μM) in serum-free medium for indicated time point. The resulting cells were collected for RNA extracting and sequencing. **(B)** HEK293T cells were disposed with serum-free medium and then treated with different concentrations of $CuSO_4$ for indicated time point. The cells were collected and lysed for immunoblot (IB) analyses (top panel). The results were normalized and analyzed using student *t* test (mean+/-SD, n = 3) (*P < 0.05, **P < 0.01, ***P < 0.001, and ****P < 0.0001) (bottom panel). **(C)** 4T1 cells were cultured in serum-free medium for 16 hours. After pretreated with TTM (100 μM) for 1 hour, the cells were disposed with $CuSO_4$ (50 μM) for different time points and then subjected to IB analysis

(left panel). The results were normalized and analyzed using student *t* test (mean+/-SD, n = 3) (*P < 0.05, **P < 0.01, ***P < 0.001, and ****P < 0.0001) (right panel). **(D-E)** BT-549 (D) and MDA-MB-468 (E) cells were cultured in serum-free medium for 16 hours and then pretreated with TTM (100 μM) for 1 hour, respectively. Next, the cells were disposed with different concentrations of $CuSO_4$ for 1hour. The resulting cells were subjected to IB analyses. **(F-G)** *CTR1* knock-out (*$Ctr1^{-/-}$)* and counterpart (*$Ctr1^{+/+}$*) MEFs cells were disposed with different concentrations of $CuSO_4$ for 1 hour and then subjected to IB analysis (F). The results were normalized and analyzed using student *t* test (mean+/-SD, n = 3) (*P < 0.05, **P < 0.01, ***P < 0.001, and ****P < 0.0001) (G). **(H)** The resulting cells from main figure 1J were subjected to cell proliferation. The results were normalized and plotted.

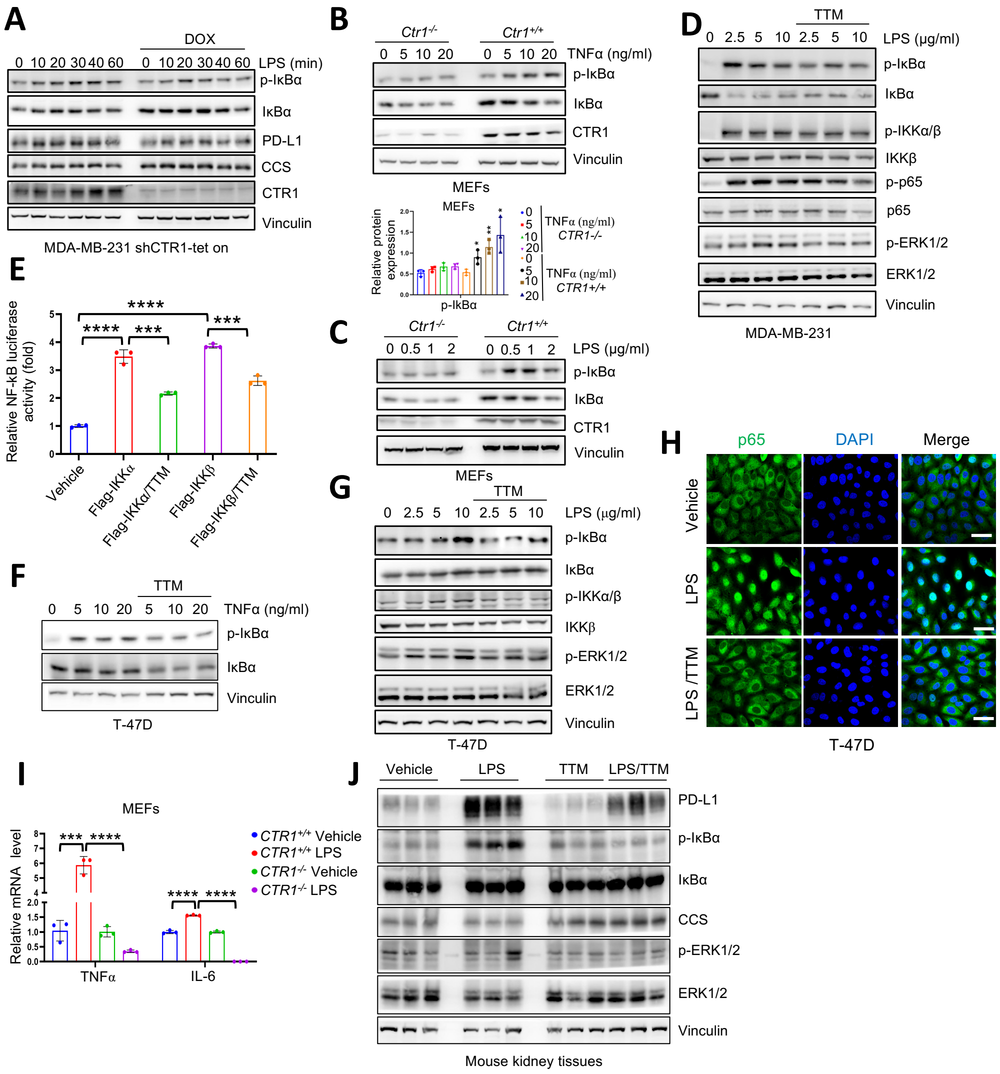

Figure S2. Copper plays a vital role in TNFα/LPS-induced NF-κB activation and PD-L1 expression

(A) MDA-MB-231 shCTR1-tet on cells were treated with or without doxycycline (1 μg/ml) for 72 hours and then the cells were disposed with LPS (5 μg/ml) for diverse time points. The resulting cells were harvested for IB analysis. **(B)** *Ctr1*$^{-/-}$ and counterpart MEFs cells were treated with different concentrations of TNFα for 1 hour and then subjected to IB analysis (top panel). The results were normalized and analyzed using student *t* test (mean+/-SD, n = 3) (*P < 0.05, **P < 0.01, ***P < 0.001, and ****P < 0.0001) (bottom panel). **(C)** *Ctr-/-* and counterpart MEFs cells were treated with different concentrations of LPS for 14 hours and then subjected to IB analysis. **(D)** MDA-MB-231 cells were pretreated with TTM (100 μM) for 1 hour and then disposed with different concentrations of LPS for 1 hour and then subjected to IB analysis. **(E)** HEK293T cells were transfected with NF-κB luciferase reporter plasmid and indicated constructs and then disposed with TTM (50 μM) for 15 hours. The cells were collected and lysed for the detection of luciferase activity. The results were analyzed using student *t* test (mean+/-SD, n = 3) (*P < 0.05,

P < 0.01, ***P < 0.001, and ****P < 0.0001). **(F-G) T-47D cells were pretreated with TTM (100 μM) for 1 hour and then disposed with different concentrations of LPS (E) and TNFα (F) for 1 hour and then subjected to IB analyses. **(H)** T-47D cells were pretreated with TTM (100 μM) for 1 hour and then disposed with LPS (10 μg/ml) for 1 hour. The cells were fixed, permeated and stained for IF analysis. Bar indicates 50 μm. **(I)** *Ctr1*$^{-/-}$ and counterpart MEFs cells were disposed with LPS (1 μg/ml) for 16 hours. The cells were collected for RNA extraction and then subjected to qRT-PCR analysis. The results were analyzed using student *t* test (mean+/-SD, n = 3) (*P < 0.05, **P < 0.01, ***P < 0.001, and ****P < 0.0001). **(J)** The female C57BL/6 mice were pretreated with indicated concentration of TTM for indicated time point and then disposed with LPS or/and TTM. After 6 hours, the kidney tissues of some mice were collected and utilized for IB analysis.

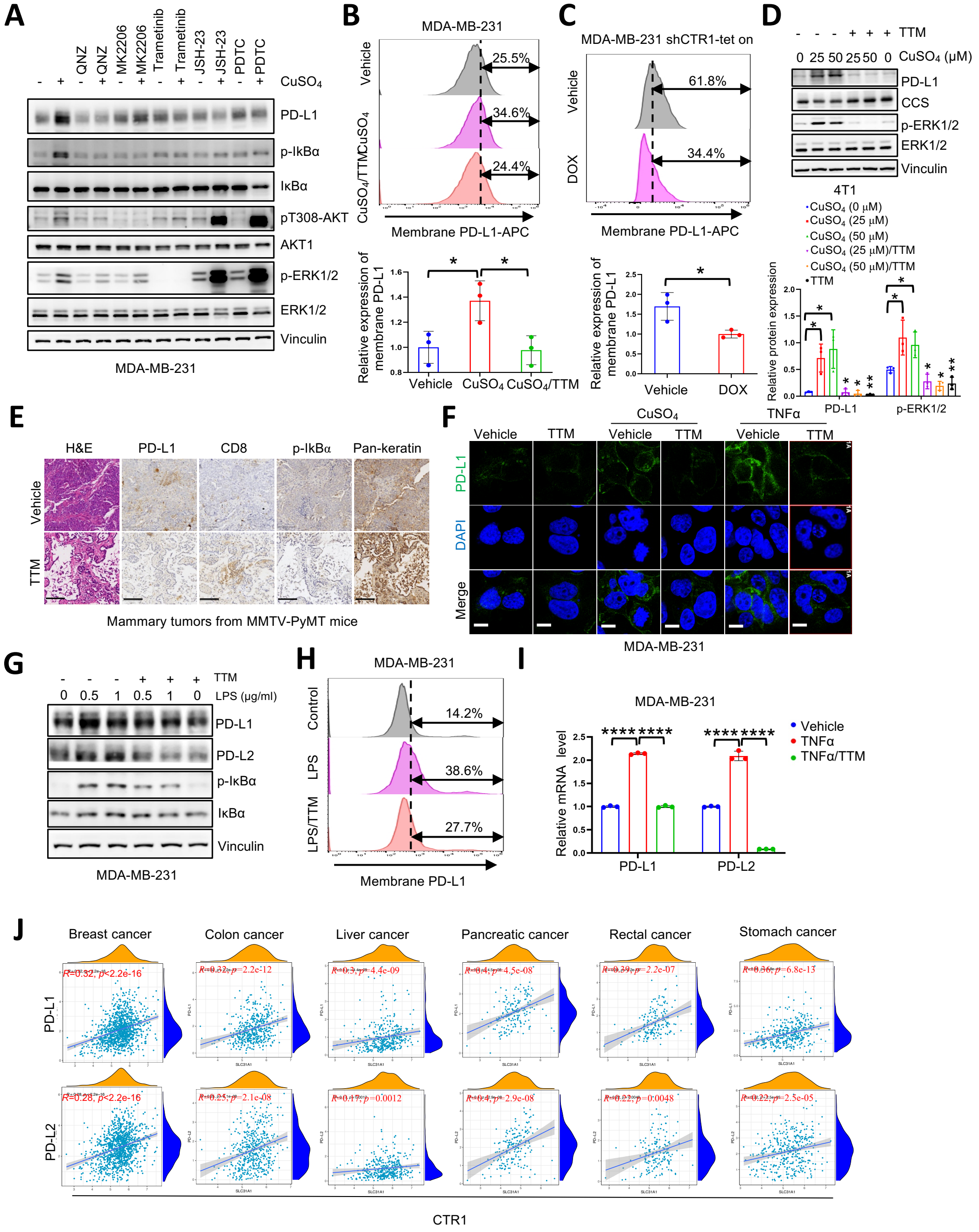

Figure S3. Copper activates NF-κB signaling to boost PD-L1 expression

(A) MDA-MB-231 cells were pretreated with QNZ (10 μM), MK-2206 (2 μM), Trametinib (1 μM), JSH-23(20 μM), PDTC (2 μM) for 1 hour in serum-free medium, respectively, then disposed with $CuSO_4$ (50 μM) for 6 hours. The resulting cells were collected for IB analysis. **(B)** MDA-MB-231 cells were disposed in serum-free medium with $CuSO_4$ (50 μM) individually or in combination with TTM (50 μM) for 14 hours and then collected for flow cytometry analysis (top panel). The results were analyzed (bottom panel) using student *t* test (mean+/-SD, n = 3) (*P < 0.05, **P < 0.01, ***P < 0.001, and ****P < 0.0001). **(C)** MDA-MB-231 shCTR1-tet on cells

were treated with doxycycline (1 μg/ml) for 72 hours and then collected for flow cytometry analysis (top panel). The results were analyzed (bottom panel) using student *t* test (mean+/-SD, n = 3) (*P < 0.05, **P < 0.01, ***P < 0.001, and ****P < 0.0001). **(D)** 4T1 cells were disposed in serum-free medium with distinct concentrations of $CuSO_4$ individually or in combination with TTM (50 μM) for indicated time point. The cells were harvested for IB analysis (top panel). The results were normalized and analyzed using student *t* test (mean+/-SD, n = 3) (*P < 0.05, **P < 0.01, ***P < 0.001, and ****P < 0.0001) (bottom panel). **(E)** The female MMTV-PyMT mice were individually treated with TTM. The mammary tumors were collected and utilized for hematoxylin and eosin (H&E) and immunohistochemistry (IHC) staining analyses. Bar indicates 100 μm. **(F)** MDA-MB-231 cells were pretreated with TTM (100 μM) for 1 hour and then disposed with $CuSO_4$ (50 μM) and TNFα (10 ng/ml) for 8 hours. The cells were fixed, permeated and subjected for IF staining. Bar indicates 10 μm. **(G)** MDA-MB-231 cells were pretreated with TTM (100 μM) for 1 hour and then disposed with different concentrations of LPS for 5 hours. The cells were subjected to IB analysis. **(H)** MDA-MB-231 cells were pretreated with TTM (100 μM) for 1 hour and then disposed with LPS (1 μg/ml) for 5 hours. The cells were collected for flow cytometry analysis. **(I)** MDA-MB-231 cells were treated with indicated concentration of TNFα (10 ng/ml) individually or in combination with TTM (50 μM) for 16 hours. The cells were collected for RNA extraction and then subjected to qRT-PCR analysis. The results were analyzed using student *t* test (mean+/-SD, n = 3) (*P < 0.05, **P < 0.01, ***P < 0.001, and ****P < 0.0001). **(J)** The correlation of CTR1 and PD-L1 expression in various cancers was analyzed with the website tool (http://gepia.cancer-pku.cn/).

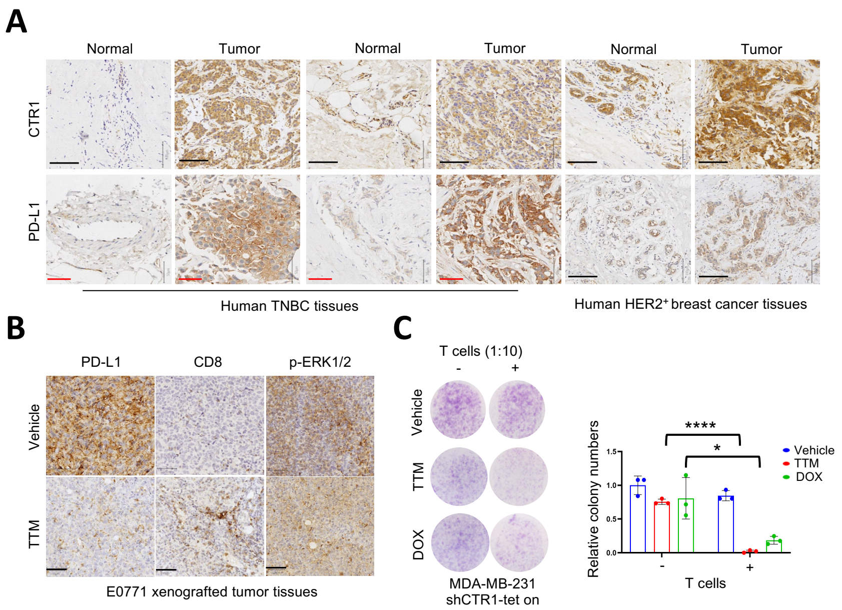

Figure S4. Decreasing copper enhances the sensitivity of T cells to breast cancer cells
(A) The sections of human TNBC tissues and normal breast tissues were stained with indicated antibodies for IHC analyses. Red bar indicates 50 µm. Black bar indicates 100 µm. **(B)** The sections of E0771 xenografts administrated with TTM and control xenografts were stained with indicated antibodies for IHC analyses. Bar indicates 50 µm. **(C)** MDA-MB-shCTR1-tet on cells were induced with or without doxycycline (1 µg/ml) for 72 hours. Meanwhile, control cells were pretreated with TTM (50 µM) for 8 hours. Then the medium was removed and the cells were washed three times using the fresh medium. Following, the cells (10000 cells/ each group) above were co-cultured with purified and activated T cells for 3 days. The obvious colonies were stained with crystal violet (left panel) and the colony numbers were analyzed (right panel) using student *t* test (mean+/-SD, n = 3). (*P < 0.05, **P < 0.01, ***P < 0.001, and ****P < 0.0001).

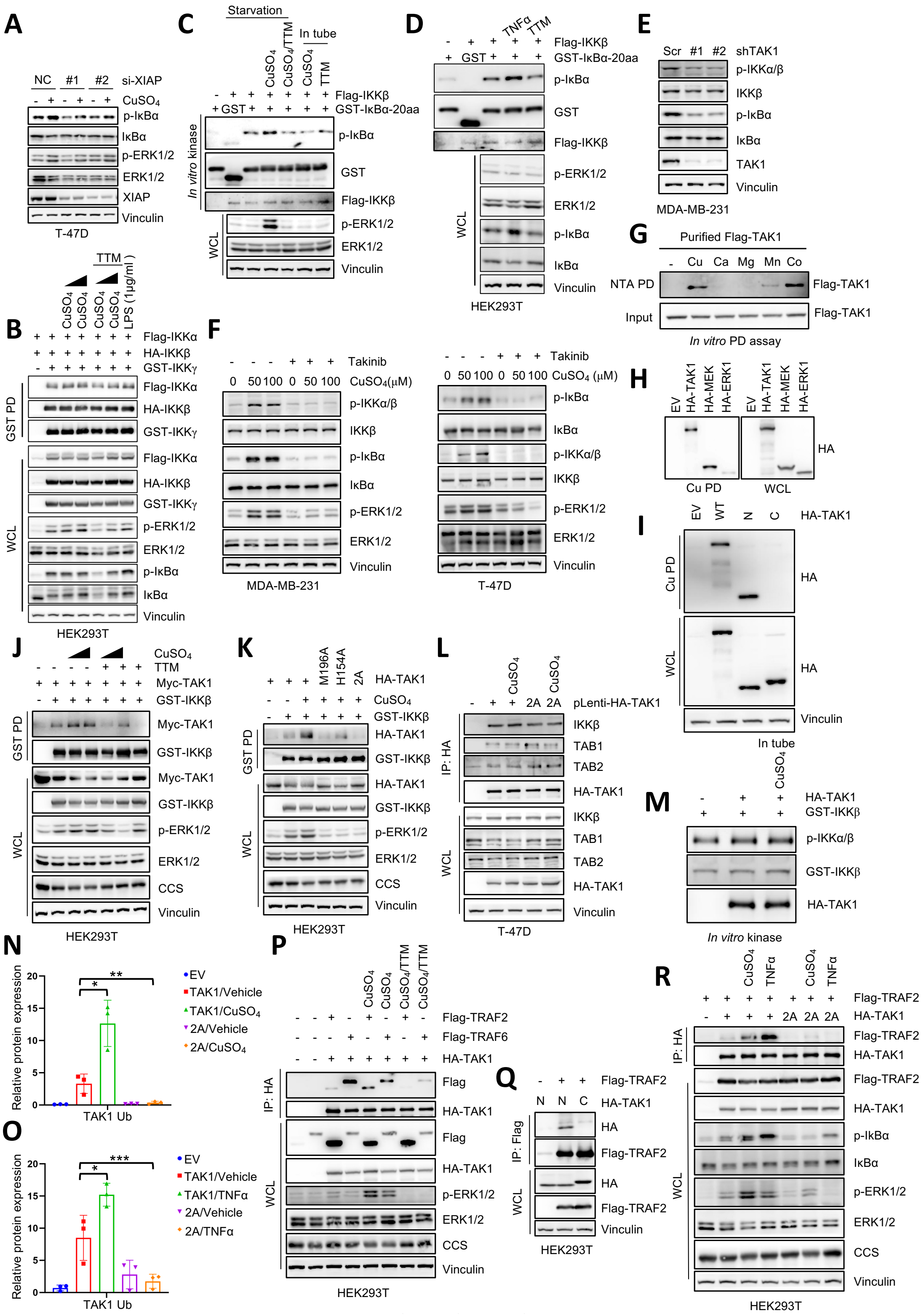

A
NC
#1
#2
si-XIAP
$CuSO_4$
p-IκBα
IκBα
p-ERK1/2
ERK1/2
XIAP
Vinculin
T-47D
B
TTM
$CuSO_4$
LPS (1μg/ml)
Flag-IKKα
HA-IKKβ
GST-IKKγ
GST PD
WCL
p-ERK1/2
ERK1/2
p-IκBα
IκBα
Vinculin
HEK293T
C
Starvation
In tube
$CuSO_4$/TTM
TTM
Flag-IKKβ
GST
GST-IκBα-20aa
In vitro kinase
p-IκBα
Flag-IKKβ
WCL
D
TNFα
GST-IκBα-20aa
HEK293T
E
Scr
shTAK1
p-IKKα/β
IKKβ
TAK1
MDA-MB-231
F
Takinib
$CuSO_4$(μM)
0
50
100
MDA-MB-231
G
Purified Flag-TAK1
Cu
Ca
Mg
Mn
Co
NTA PD
Input
Flag-TAK1
In vitro PD assay
H
EV
HA-TAK1
HA-MEK
HA-ERK1
HA
Cu PD
I
WT
N
C
In tube
J
TTM
Myc-TAK1
GST-IKKβ
CCS
K
M196A
H154A
2A
HA-TAK1
L
pLenti-HA-TAK1
IP: HA
TAB1
TAB2
M
In vitro kinase
N
Relative protein expression
TAK1 Ub
EV
TAK1/Vehicle
TAK1/$CuSO_4$
2A/Vehicle
2A/$CuSO_4$
O
TAK1/TNFα
2A/TNFα
P
Flag-TRAF2
Flag-TRAF6
Flag
Q
IP: Flag
R

Figure S5. Copper activates NF-κB signaling through TAK1

(A) T-47D cells were transfected with small interfering RNAs targeting *XIAP* for 72 hours and then were treated with $CuSO_4$ (50 μM) for 2 hours after disposed in serum-free medium for 16 hours. The resulting cells were collected and subjected to IB analysis. **(B)** HEK293T cells were transfected with indicated constructs and then disposed in serum-free medium and different concentrations of $CuSO_4$ (25 μM and 50 μM) individually or in combination with TTM (50 μM) and LPS for 14 hours. The cells were harvested for GST pull-down and IB analysis. **(C)** HEK293T cells were transfected with indicated constructs and then disposed using $CuSO_4$ (100 μM) individually or in combination with TTM (100 μM) for 40 mins after cultivated in serum-free medium for 16 hours, following the kinase IKKβ was immuno-precipitated from cells above. The substrate IkBα-20aa was purified from bacteria. The indicated IKKβ and IkBα-20aa were added into the kinase buffer in the presence or absence of $CuSO_4$ (100 μM) and TTM (100 μM). The results were analyzed via IB assay. **(D)** HEK293T cells were transfected with indicated constructs and then disposed with TNFα (10 ng/ml) or TTM (100 μM) for 40 mins, following the kinase IKKβ was immuno-precipitated from cells above. The substrate IκBα-20aa was purified from bacteria. The indicated IKKβ and IκBα-20aa were added into the kinase buffer and the products were analyzed via IB assay. **(E)** MDA-MB-231 cells were infected with lentiviruses encoding different TAK1 shRNAs and then selected with puromycin (1 μg/ml). The resulting cells were subjected to IB analysis. **(F)** MDA-MB-231 (left panel) and T-47D (right panel) cells were cultured in serum-free medium for 16 hours, and then pretreated with TAK1 inhibitor Takinib (10 μM) for 1 hour. Following, the cells were treated with $CuSO_4$ (50 μM and 100 μM) for 1 hour and then subjected to IB analyses. **(G)** The protein Flag-TRAF2 was purified using 3 x Flag peptide and then were respectively incubated with NTA beads containing different divalent metals for 0.5 hour at 4 ^{0}C and then the beads were washed using NETN buffer (20 mM Tris-HCl (PH 8.0), 300 mM NaCl, 0.5% NP-40, 1 mM EDTA) for 4 times and subjected to IB analysis. **(H)** HEK293T cells were transfected with indicated constructs for indicated time point. The resulting cells were lysed and then subjected to copper pull-down and IB analysis. **(I)** Wild-type TAK1 and the plasmid containing N-terminal 300 amino acid fragment or C-terminal 306 amino acid fragment of TAK1 were transfected into HEK293T cells. The resulting cells were collected and then subjected to copper pull-down (PD) assay. **(J)** HEK293T cells were transfected with indicated constructs, then cultivated in serum-free medium and disposed with distinct concentrations of $CuSO_4$ (25 μM and 50 μM) individually or in combination with TTM (50 μM) for 14 hours. The resulting cells were harvested and subjected to GST pull-down (PD) and IB analyses. **(K)** HEK293T cells were transfected with indicated constructs and then cultivated in serum-free medium and disposed with $CuSO_4$ (50 μM) for 14 hours. The resulting cells were subjected to GST pull-down (PD) and IB analyses. **(L)** T-47D cells stably expressing TAK1 and the mutant 2A were cultured in in serum-free medium for 16 hours and then disposed with $CuSO_4$ (50 μM) for 14 hours. The resulting cells were harvested and subjected to anti-HA IP analysis. **(M)** HEK293T cells were transfected with indicated constructs and then the kinase TAK1 was immuno-precipitated from cells above. The substrate IKKβ was immuno-precipitated and purified from HEK293T cells transfected with indicated construct. The indicated TAK1 and IKKβ were

added into the kinase buffer in the presence or absence of $CuSO_4$ (100 μM) and the products were subjected to IB analysis. **(N-O)** Quantitative analyses of protein expression for main figure 3K (N) and 3L (O). The results were analyzed using student *t* test (mean+/-SD, n = 3) (*P < 0.05, **P < 0.01, ***P < 0.001, and ****P < 0.0001). **(P)** The plasmids Flag-TRAF2, Flag-TRAF6 and HA-TAK1 were cotransfected into HEK293T cells. Following, the cells were disposed in serum-free medium with $CuSO_4$ (50 μM) and/or TTM (50 μM) for 12 hours. The resulting cells were harvested and then subjected to anti-HA IP analysis. **(Q)** The plasmids wild-type TAK1, mutant TAK1 (N-terminal fragment containing 300 amino acids or C-terminal fragment containing 306 amino acids) and Flag-TRAF2 were cotransfected into HEK293T cells. The resulting cells were subjected to anti-Flag IP analysis. **(R)** The plasmids wild-type TAK1, mutant TAK1 (2A) and Flag-TRAF2 were cotransfected into HEK293T cells. Next, the cells were disposed in serum-free medium with $CuSO_4$ (50 μM) for 12 hours and TNFα (20 ng/ml) for 0.5 hour, respectively. The resulting cells were subjected to IB analysis.

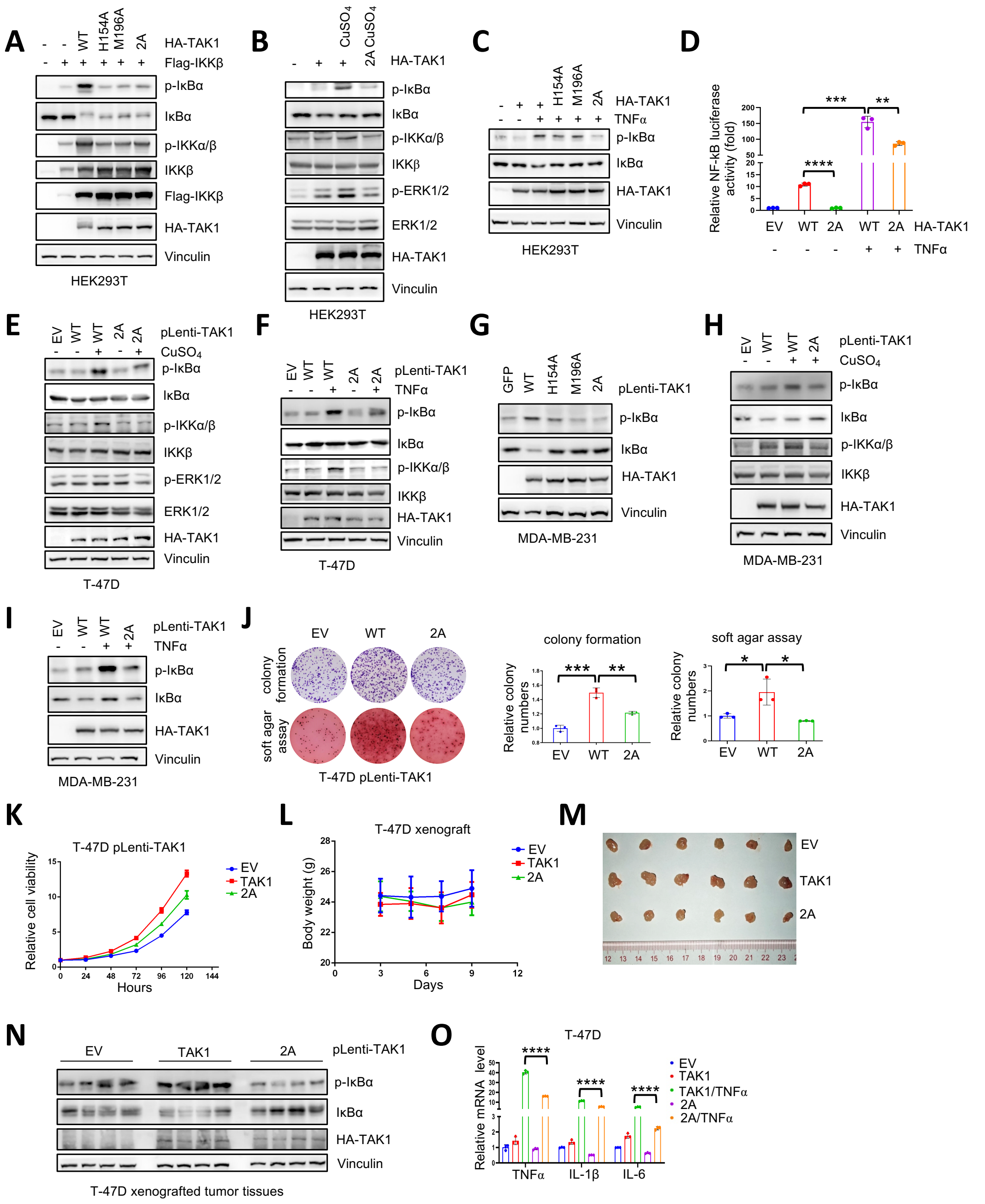

Figure S6. Copper activates NF-κB signaling through TAK1

(A) HEK293T cells were transfected with indicated constructs and then subjected to IB analysis. **(B)** HEK293T cells were transfected with indicated constructs containing TAK1 or corresponding mutant 2A and then disposed with $CuSO_4$ (50 μM) for 1 hour after cultivated in serum-free medium for 16 hours and subsequently subjected to IB analysis. **(C)** HEK293T cells were transfected with indicated constructs containing TAK1 or corresponding mutants and then disposed with TNFα (20 ng/ml) for 1 hour. The resulting cells were subjected to IB analysis. **(D)** HEK293T cells were transfected with NF-κB luciferase reporter plasmid and indicated constructs.

Following, the cells above were disposed with TNFα (10 ng/ml) for 16 hours. The cells were collected and lysed for the detection of luciferase activity. The results were analyzed using student *t* test (mean+/-SD, n = 3) (*P < 0.05, **P < 0.01, ***P < 0.001, ****P < 0.0001). **(E-F)** T-47D cells were infected with lentiviruses encoding TAK1 and corresponding mutant 2A and then selected with hygromycin (100 μg/ml). The resulting cells were disposed with $CuSO_4$ (50 μM) after cultured in serum-free medium for 16 hours (E) or stimulated with TNFα (20 ng/ml) (F) for 1 hour, subsequently subjected to IB analyses. **(G)** MDA-MB-231 cells were infected with lentiviruses encoding TAK1 or corresponding mutant 2A and then selected with hygromycin (100 μg/ml) and subsequently subjected to IB analysis. **(H-I)** MDA-MB-231 cells stably expressing wild-type or mutant TAK1 were disposed with TNFα (20 ng/ml) (H) or $CuSO_4$ (50 μM) after cultured in serum-free medium for 16 hours (I) for 1 hour, subsequently subjected to IB analyses. **(J)** T-47D cells were infected with lentiviruses encoding wild-type or mutant TAK1 and then selected with hygromycin (100 μg/ml). The resulting cells were subjected to colony formation and soft agar assay (left panel). Relative colony numbers were normalized and plotted (right panel). The results were analyzed using student *t* test (mean+/-SD, n = 3) (*P < 0.05, **P < 0.01, ***P < 0.001, and ****P < 0.0001). **(K)** Cell viabilities of T-47D cells stably expressing wild-type or mutant TAK1 were detected and analyzed for different time points. **(L-N)** T-47D cells stably expressing wild-type or mutant TAK1 were subjected to xenograft assay. The mouse weight (L) was monitored (mean+/-SD, n = 6) (*P < 0.05, **P < 0.01, ***P < 0.001, and ****P < 0.0001, *ANOVA* test). The tumors were dissected (M). Small portion of T-47D xenografts were subjected to IB analysis (N). **(O)** T-47D cells stably expressing wild-type or mutant TAK1 were treated in the presence or absence of TNFα (5 ng/ml) for 16 hours. The resulting cells were collected for RNA extraction and then subjected to qRT-PCR analysis. The results were analyzed using student *t* test (mean+/-SD, n = 3) (*P < 0.05, **P < 0.01, ***P < 0.001, and ****P < 0.0001).

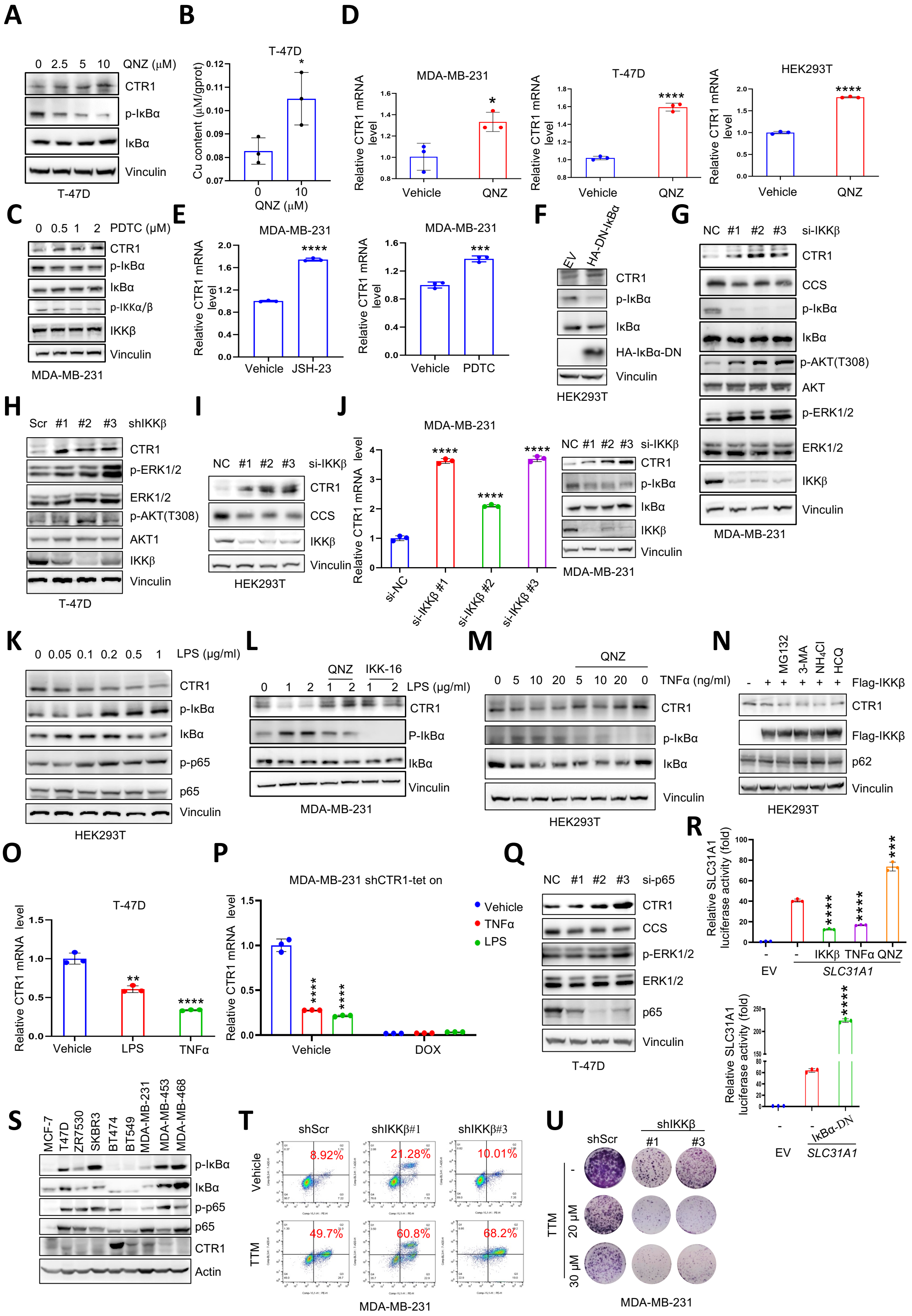
A
0 2.5 5 10 QNZ (μM)
CTR1
p-IκBα
IκBα
Vinculin
T-47D
B
T-47D
Cu content (μM/gprot)
0 10
QNZ (μM)
D
MDA-MB-231
T-47D
HEK293T
Relative CTR1 mRNA level
Vehicle QNZ
C
0 0.5 1 2 PDTC (μM)
CTR1
p-IκBα
IκBα
p-IKKα/β
IKKβ
Vinculin
MDA-MB-231
E
MDA-MB-231
Relative CTR1 mRNA level
Vehicle JSH-23
Vehicle PDTC
F
EV HA-DN-IκBα
CTR1
p-IκBα
IκBα
HA-IκBα-DN
Vinculin
HEK293T
G
NC #1 #2 #3 si-IKKβ
CTR1
CCS
p-IκBα
IκBα
p-AKT(T308)
AKT
p-ERK1/2
ERK1/2
IKKβ
Vinculin
MDA-MB-231
H
Scr #1 #2 #3 shIKKβ
CTR1
p-ERK1/2
ERK1/2
p-AKT(T308)
AKT1
IKKβ
Vinculin
T-47D
I
NC #1 #2 #3 si-IKKβ
CTR1
CCS
IKKβ
Vinculin
HEK293T
J
MDA-MB-231
Relative CTR1 mRNA level
si-NC
si-IKKβ #1
si-IKKβ #2
si-IKKβ #3
NC #1 #2 #3 si-IKKβ
CTR1
p-IκBα
IκBα
IKKβ
Vinculin
MDA-MB-231
K
0 0.05 0.1 0.2 0.5 1 LPS (μg/ml)
CTR1
p-IκBα
IκBα
p-p65
p65
Vinculin
HEK293T
L
QNZ IKK-16
0 1 2 1 2 1 2 LPS (μg/ml)
CTR1
P-IκBα
IκBα
Vinculin
MDA-MB-231
M
QNZ
0 5 10 20 5 10 20 0 TNFα (ng/ml)
CTR1
p-IκBα
IκBα
Vinculin
HEK293T
N
MG132 3-MA NH4Cl HCQ
- + + + + + Flag-IKKβ
CTR1
Flag-IKKβ
p62
Vinculin
HEK293T
O
T-47D
Relative CTR1 mRNA level
Vehicle LPS TNFα
P
MDA-MB-231 shCTR1-tet on
Relative CTR1 mRNA level
Vehicle
TNFα
LPS
Vehicle DOX
Q
NC #1 #2 #3 si-p65
CTR1
CCS
p-ERK1/2
ERK1/2
p65
Vinculin
T-47D
R
Relative SLC31A1 luciferase activity (fold)
- - IKKβ TNFα QNZ
EV SLC31A1
Relative SLC31A1 luciferase activity (fold)
- - IκBα-DN
EV SLC31A1
S
MCF-7 T47D ZR7530 SKBR3 BT474 BT549 MDA-MB-231 MDA-MB-453 MDA-MB-468
p-IκBα
IκBα
p-p65
p65
CTR1
Actin
T
shScr shIKKβ#1 shIKKβ#3
Vehicle
TTM
8.92%
21.28%
10.01%
49.7%
60.8%
68.2%
MDA-MB-231
U
shScr shIKKβ
#1 #3
TTM
-
20 μM
30 μM
MDA-MB-231

Figure S7. NF-κB activation represses CTR1 expression

(A) T-47D cells were treated with different concentrations of QNZ for 14 hours, respectively. The resulting cells were collected and subjected to IB analysis. **(B)** T-47D cells were disposed with QNZ (10 μM) for 14 hours and then collected for measuring the copper content. The results were normalized and analyzed using student *t* test (mean+/-SD, n = 3) (*P < 0.05, **P < 0.01, ***P < 0.001, and ****P < 0.0001). **(C)** MDA-MB-231 cells were treated with different concentrations of NF-κB inhibitor PDTC for 12 hours and the subjected to IB analysis. **(D)** MDA-MB-231, T-47D and HEK293T cells were disposed with QNZ (10 μM) for 16 hours and then collected for RNA extraction and then subjected to qRT-PCR analyses. The results were analyzed using student *t* test (mean+/-SD, n = 3) (*P < 0.05, **P < 0.01, ***P < 0.001, and ****P < 0.0001). **(E)** MDA-MB-231 cells were treated with NF-κB inhibitors JSH-23 (10 μM) and PDTC (0.5 μM) for 14 hours and then collected for RNA extraction and then subjected to qRT-PCR analyses. The results were analyzed using student *t* test (mean+/-SD, n = 3) (*P < 0.05, **P < 0.01, ***P < 0.001, and ****P < 0.0001). **(F)** HEK293T cells were transfected with indicated construct containing DN-IκBα and then subjected to IB analysis. **(G)** MDA-MB-231 cells were transfected with small interfering RNAs targeting *IKKβ* for 72 hours. The resulting cells were collected and subjected to IB analysis. **(H)** T-47D cells were infected with shRNA lentiviruses targeting *IKKβ* and then selected with puromycin (1 μg/ml). The resulting cells were collected and subjected to IB analysis. **(I)** HEK293T cells were transfected with small interfering RNAs targeting *IKKβ* for 72 hours. The resulting cells were collected and subjected to IB analysis. **(J)** MDA-MB-231 cells were transfected with small interfering RNAs targeting *IKKβ* for 72 hours. The resulting cells were collected and subjected to qRT-PCR (left panel) and IB (right panel) analyses. The results of qRT-PCR were analyzed using student *t* test (mean+/-SD, n = 3) (*P < 0.05, **P < 0.01, ***P < 0.001, and ****P < 0.0001). **(K)** HEK293T cells were disposed with different concentrations of LPS and then subjected to IB analysis. **(L)** MDA-MB-231 cells were pretreated with QNZ (10 μM) or IKKβ inhibitor IKK-16 (0.6 μM) for 1 hour and stimulated with distinct concentrations of LPS for 14 hours and then subjected to IB analysis. **(M)** HEK293T cells were pretreated with QNZ (10 μM) for 1 hour and then disposed with distinct concentrations of TNFα for indicated time points. The resulting cells were then subjected to IB analysis. **(N)** HEK293T cells were transfected with indicated construct and then treated with MG132 (30 μM), 3-MA (5 mM), NH_4Cl (10 mM), HCQ (20 μM) for 6 hours. The cells were harvested and for IB analysis. **(O)** T-47D cells were treated with LPS (1 μg/ml) and TNFα (10 ng/ml) for 16 hours. The cells were collected for RNA extraction and then subjected to qRT-PCR analyses. The results were analyzed using student *t* test (mean+/-SD, n = 3) (*P < 0.05, **P < 0.01, ***P < 0.001, and ****P < 0.0001). **(P)** MDA-MB-231 shCTR1-tet on cells were treated with or without doxycycline (1 μg/ml) for 72 hours and then the cells were disposed with TNFα (10 ng/ml) and LPS (2 μg/ml) for 16 hours. The cells were collected for RNA extraction and then subjected to qRT-PCR analysis. The results were analyzed using student *t* test (mean+/-SD, n = 3) (*P < 0.05, **P < 0.01, ***P < 0.001, and ****P < 0.0001). **(Q)** T-47D cells were transfected with small interfering RNAs targeting *p65* for 72 hours. The resulting cells were collected and subjected to IB analysis. **(R)** HEK293T cells were transfected with the luciferase reporter plasmid containing human *SLC31A1* promoter and indicated

constructs. The cells were treated with or without TNFα (20 ng/ml) and QNZ (10 μM) and then collected and lysed for the detection of luciferase activity. The results were analyzed using student *t* test (mean+/-SD, n = 3) (*P < 0.05, **P < 0.01, ***P < 0.001, and ****P < 0.0001). **(S)** Various breast cancer cells were collected and then subjected to IB analysis. **(T-U)** *IKKβ* knock-down MDA-MB-231 and control cells were disposed with different concentrations of TTM (G, 60 μM; H, 20 μM or 30 μM) for 72 hours (apoptosis assay) (T) or 14 days (colony formation) (U).

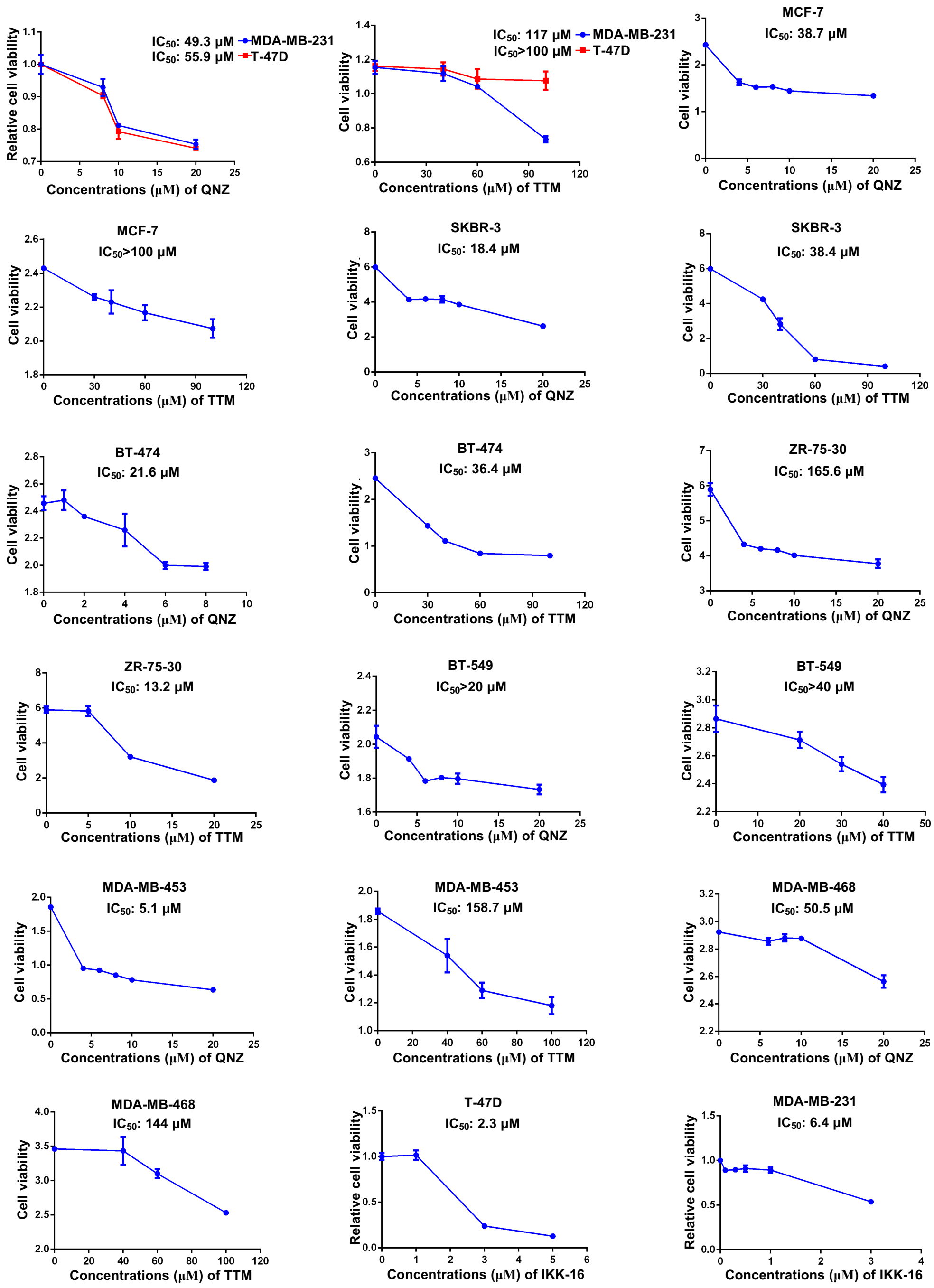

Figure S8. IC_{50} of NF-κB inhibitor or copper chelator for various breast cancer cells Various breast cancer cells were treated with different doses of NF-κB inhibitor or copper chelator TTM for indicated time points, the viabilities of resulting cells were measured and analyzed using GraphPad Prism version 6.0. IC_{50} value of QNZ or TTM was calculated using SPSS statistic 19.0. The number of replicates was 3 times and the error bars represent standard deviation.

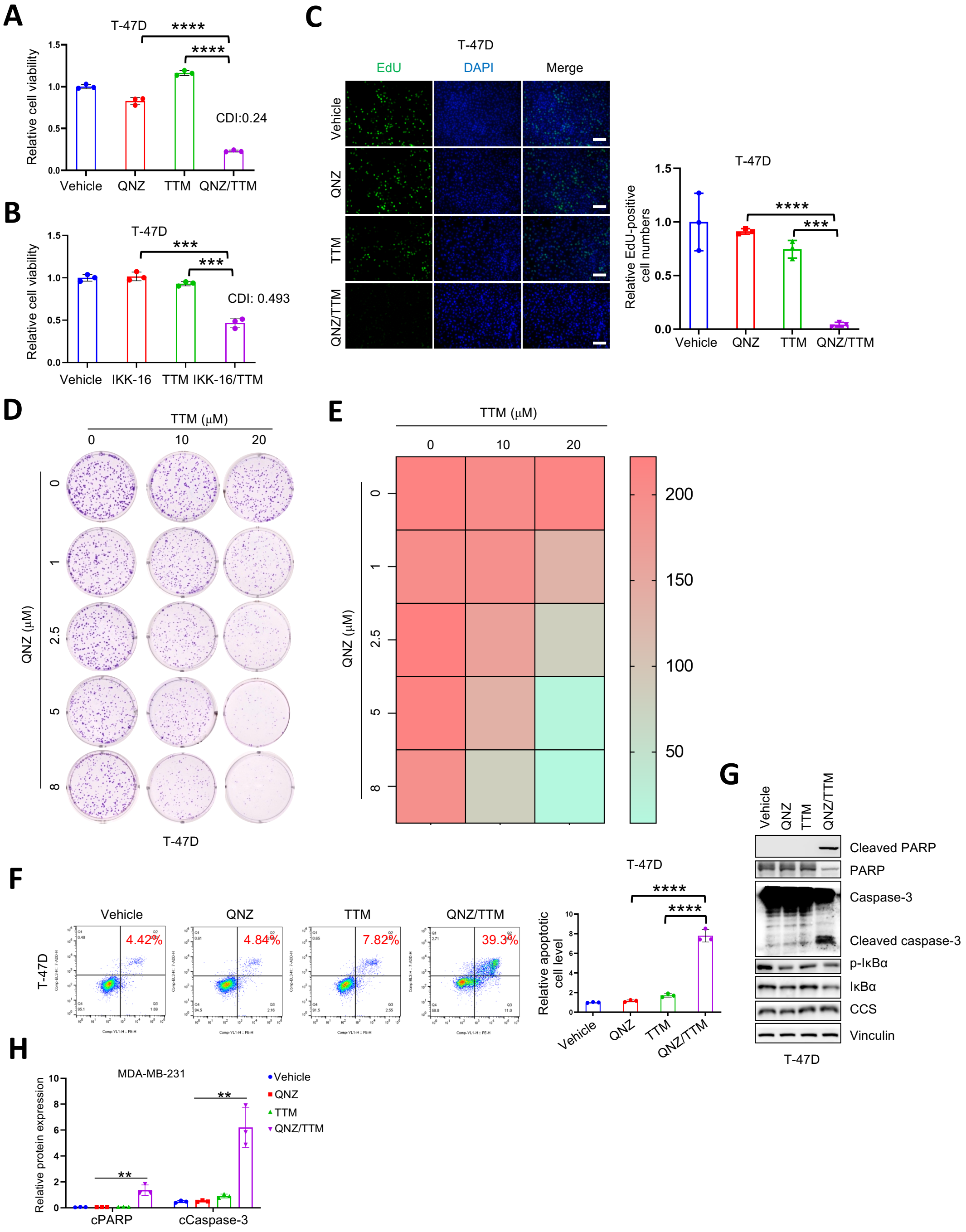

Figure S9. NF-κB inhibitor synergizes with copper chelator in breast cancer cells

(A-B) T-47D cells were treated with indicated concentrations of QNZ (A, 8 μM) or IKK-16 (B, 1 μM) and TTM (30 μM) individually or in combination for 24 hours, then the cell viabilities were detected and analyzed using student *t* test (mean+/-SD, n = 3) (*P < 0.05, **P < 0.01, ***P < 0.001, and ****P < 0.0001). Coefficient of drug in interaction (CDI) was calculated. CDI<1 means synergistic effect, CDI<0.7 means significantly synergistic effect. **(C)** T-47D cells were

treated with QNZ (5 μM) and TTM (100 μM) individually or in combination for 12 hours, and the resulting cells were fixed and labeled with EdU (left panel), relative EdU-labeled cell numbers were normalized and plotted (right panel). The results were analyzed using student *t* test (mean+/-SD, n = 3) (*P < 0.05, **P < 0.01, ***P < 0.001, and ****P < 0.0001). Bar indicates 10 μm. **(D-E)** T-47D cells were treated with indicated concentrations of QNZ and TTM individually or in combination for colony formation assay. The resulting cells were fixed and stained with crystal violet solution (D) and heat map of quantified colony numbers was plotted (E). **(F-G)** T-47D cells were treated with QNZ (10 μM) and TTM (100 μM) individually or in combination for 24 hours, the resulting cells were subjected to Annexin V-PE/7-AAD-labeled apoptosis assay (F, left panel) and IB analysis (G), apoptotic cells were quantified and analyzed (F, right panel) using student *t* test (mean+/-SD, n = 3) (*P < 0.05, **P < 0.01, ***P < 0.001, and ****P < 0.0001). **(H)** Quantitative analysis of protein expression for main figure 5G. The results were analyzed using student *t* test (mean+/-SD, n = 3) (*P < 0.05, **P < 0.01, ***P < 0.001, and ****P < 0.0001).

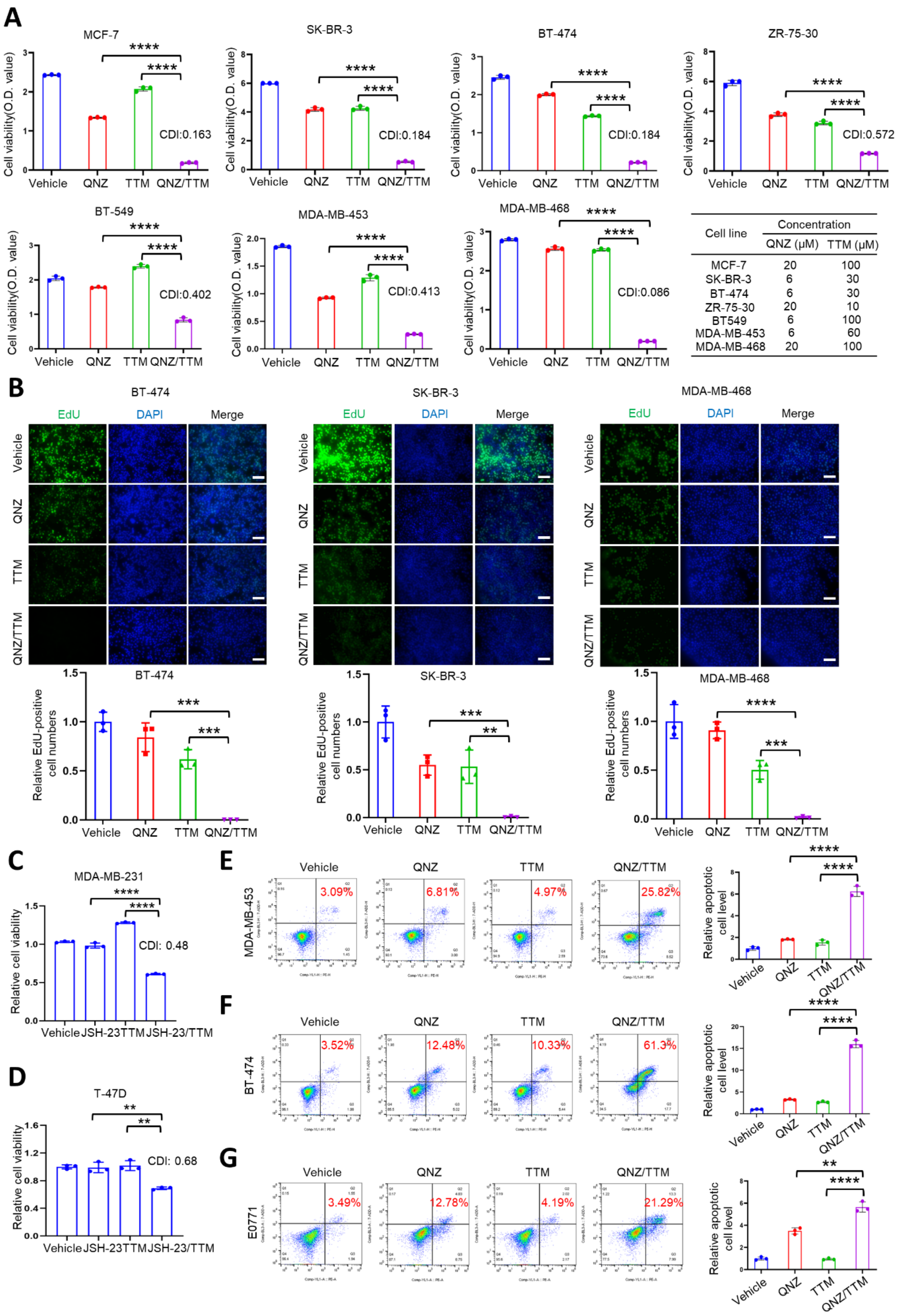

Cell line	Concentration	
	QNZ (μM)	TTM (μM)
MCF-7	20	100
SK-BR-3	6	30
BT-474	6	30
ZR-75-30	20	10
BT549	6	100
MDA-MB-453	6	60
MDA-MB-468	20	100

Figure S10. Combination of NF-κB inhibitor and copper chelator for cancer intervention in breast cancer cells

(A) Different breast cancer cells were treated with indicated concentrations of QNZ and TTM individually or in combination for 24 hours, then the cell viabilities were detected and analyzed using student *t* test (mean+/-SD, n = 3) (*P < 0.05, **P < 0.01, ***P < 0.001, and ****P < 0.0001). Coefficient of drug in interaction (CDI) was calculated. CDI<1 means synergistic effect, CDI<0.7 means significantly synergistic effect. **(B)** Various breast cancer cells were treated with indicated concentrations of QNZ and TTM individually or in combination for 12 hours, and the resulting cells were fixed and labeled with EdU (top panel), relative EdU-labeled cell numbers were normalized and plotted (bottom panel). The results were analyzed using student *t* test (mean+/-SD, n = 3) (*P < 0.05, **P < 0.01, ***P < 0.001, and ****P < 0.0001). Bar indicates 100 μm. **(C-D)** MDA-MB-231 and T-47D cells were treated with indicated concentrations of NF-κB inhibitor JSH-23 (40 μM) and TTM (MDA-MB-231, 35 μM; T-47D, 100 μM) individually or in combination for 48 hours, respectively, then the cell viabilities were detected and analyzed using student *t* test (mean+/-SD, n = 3) (*P < 0.05, **P < 0.01, ***P < 0.001, and ****P < 0.0001). Coefficient of drug in interaction (CDI) was calculated. CDI<1 means synergistic effect, CDI<0.7 means significantly synergistic effect. **(E-G)** MDA-MB-453, BT-474 and E0771 cells were treated with QNZ (E, 5 μM; F, 8 μM; G, 5 μM) and TTM (E, 40 μM; F, 30 μM; G, 10 μM) individually or in combination for 24 hours, the resulting cells were subjected to Annexin V-PE/7-AAD-labeled apoptosis assays (E-G, left panel), apoptotic cells were quantified and analyzed (F-G, right panel) using student *t* test (mean+/-SD, n = 3) (*P < 0.05, **P < 0.01, ***P < 0.001, and ****P < 0.0001).

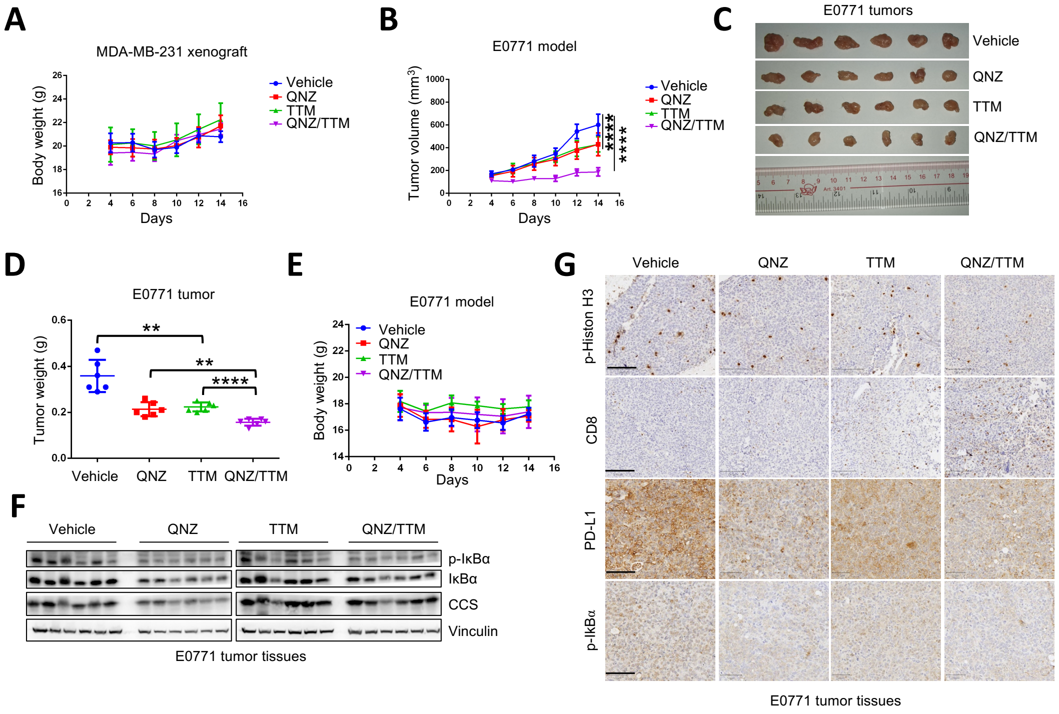

Figure S11. NF-κB inhibitor synergizes with copper chelator for breast cancer *in vivo*

(A) MDA-MB-231 cells were subjected to xenograft assay. The mouse body weight was monitored (mean+/-SD, n = 7) (*P < 0.05, **P < 0.01, ***P < 0.001, and ****P < 0.0001, *ANOVA* test). **(B-G)** The C57BL/6J mice with E0771 tumors were treated with QNZ and TTM individually or in combination. The tumor size (B) and mouse body weight (E) were monitored (mean+/-SD, n = 6) (*P < 0.05, **P < 0.01, ***P < 0.001, and ****P < 0.0001, *ANOVA* test). The tumors were dissected and weighed (C and D) (mean+/-SD, n = 6). *P < 0.05, **P < 0.01, ***P < 0.001, and ****P < 0.0001 (student *t* test). The tumors were subjected to IB analysis (F) and IHC assay (G) with indicated antibodies. Bar indicates 100 μm.

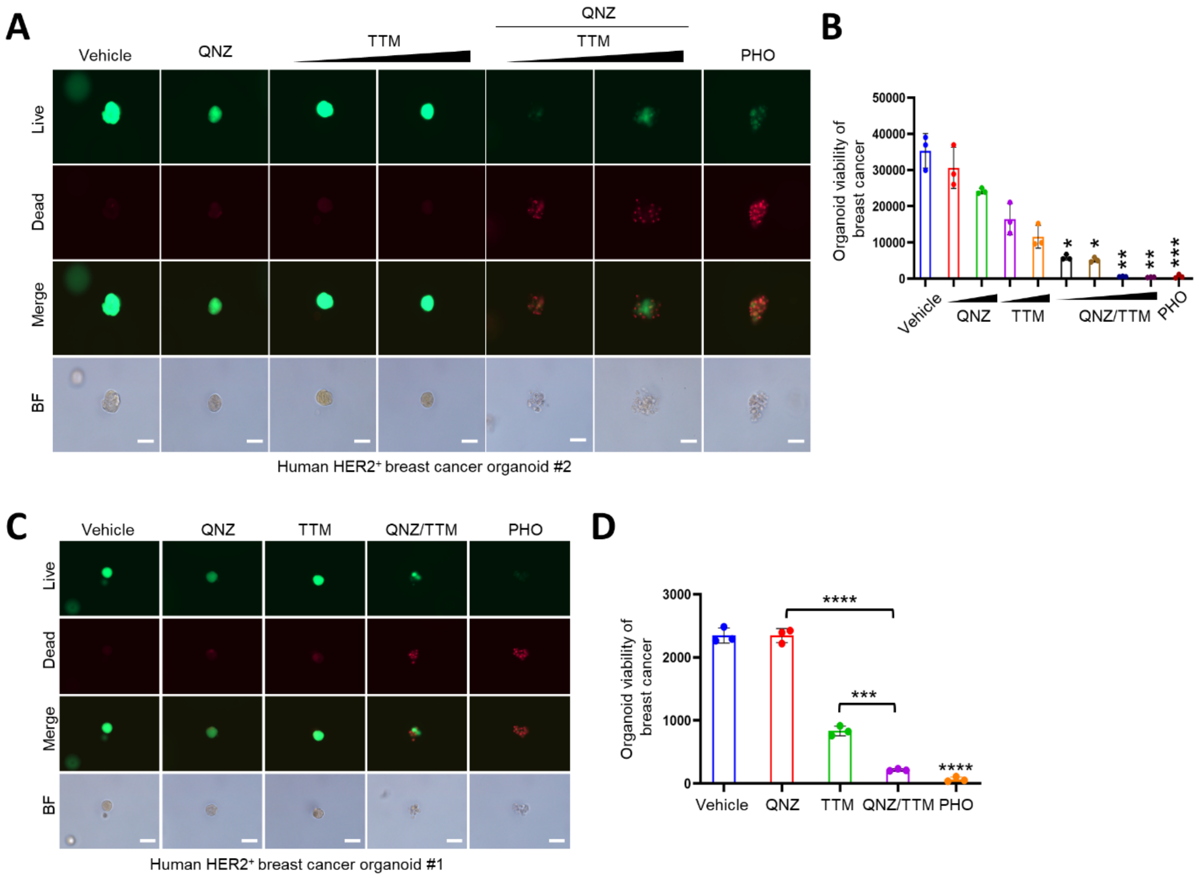

Figure S12. NF-κB inhibitor synergizes with copper chelator for breast cancer organoids (A-D) Breast cancer organoids were treated with QNZ (A, 5 μM; B, 2.5 μM, 5 μM; C and D, 10 μM) and TTM (A, 20 μM, 30 μM; B, 10 μM, 20 μM; C and D, 20 μM) individually or in combination for 48 hours, then the live and dead organoids were stained (A and C), the viabilities of organoids were detected and analyzed (B and D) using student *t* test (mean+/-SD, n = 3) (*P < 0.05, **P < 0.01, ***P < 0.001, and ****P < 0.0001). Bar indicates 50 μm.

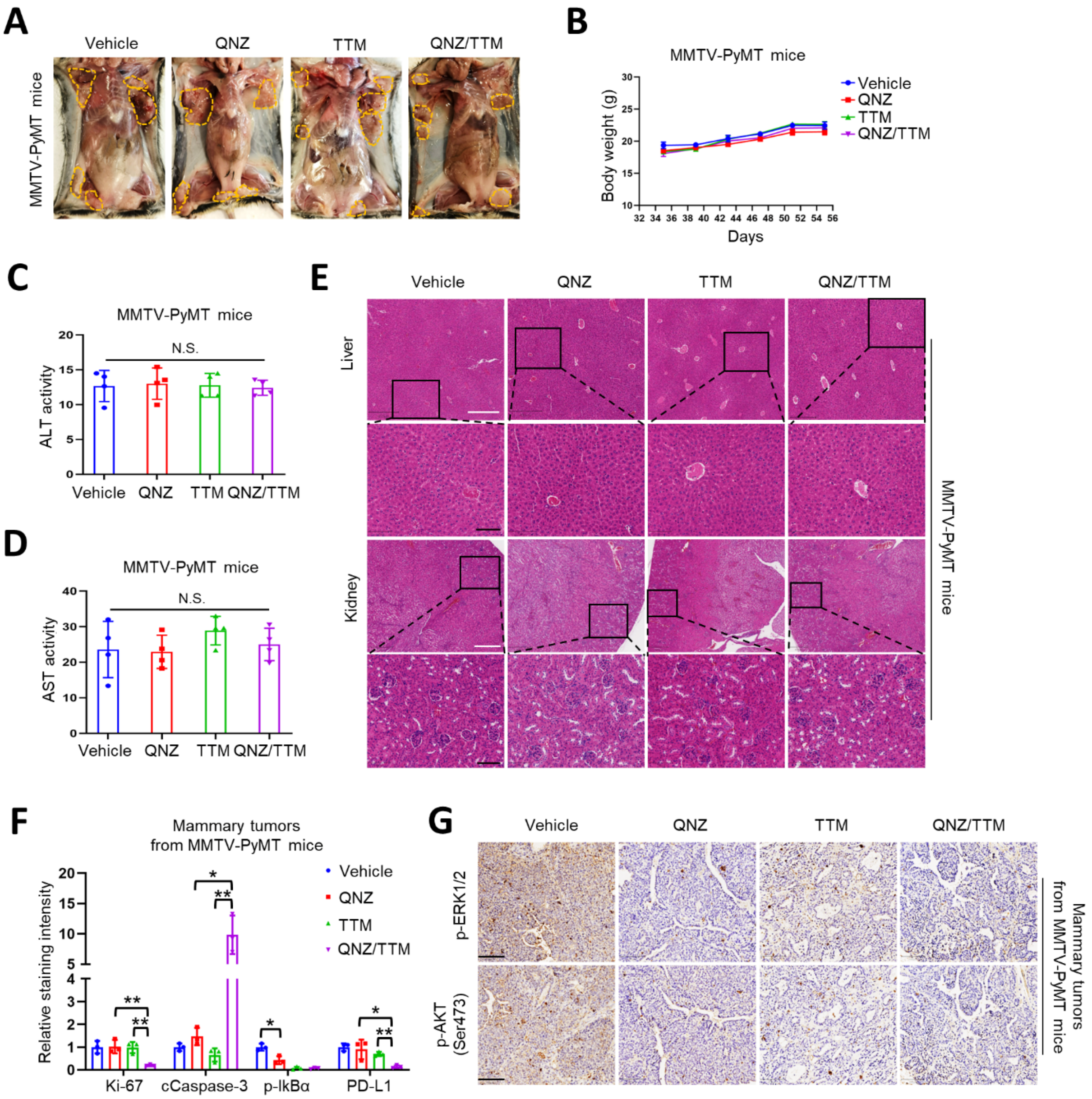

Figure S13. NF-κB inhibitor synergizes with copper chelator for MMTV-PyMT mice
(A-G) Female MMTV-PyMT mice were treated with indicated concentrations of QNZ and TTM individually or in combination for indicated time point. The mice were euthanized and then the tumors, livers and kidneys were dissected (A). The body weights of mice at different time points were recorded and analyzed (B). The toxicities of liver and kidney were evaluated by detecting ALT (C) and AST (D) activities or H&E staining (E). Quantitative analysis of IHC staining for main figure 6F (F). The results were analyzed using student *t* test or *ANOVA* test (mean+/-SD, n = 3) (*P < 0.05, **P < 0.01, ***P < 0.001, and ****P < 0.0001). The mammary tumors of mice were subjected to IHC staining assay (G) with indicated antibodies. White bar indicates 400 μm. Black bar indicates 100 μm.

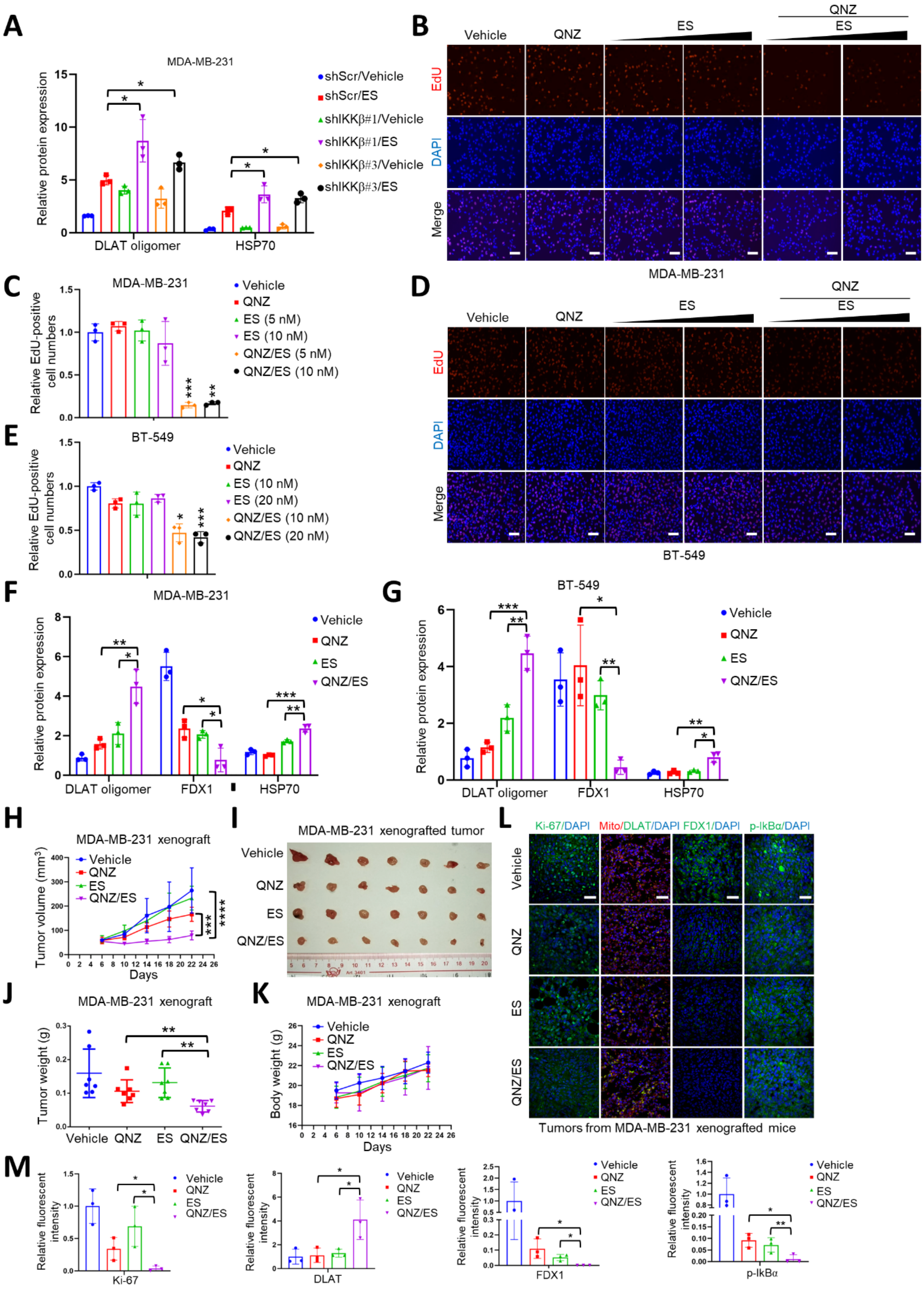
A
MDA-MB-231
Relative protein expression
DLAT oligomer
HSP70
shScr/Vehicle
shScr/ES
shIKKβ#1/Vehicle
shIKKβ#1/ES
shIKKβ#3/Vehicle
shIKKβ#3/ES
B
Vehicle
QNZ
ES
QNZ
ES
EdU
DAPI
Merge
MDA-MB-231
C
MDA-MB-231
Relative EdU-positive cell numbers
Vehicle
QNZ
ES (5 nM)
ES (10 nM)
QNZ/ES (5 nM)
QNZ/ES (10 nM)
D
Vehicle
QNZ
ES
QNZ
ES
EdU
DAPI
Merge
BT-549
E
BT-549
Relative EdU-positive cell numbers
Vehicle
QNZ
ES (10 nM)
ES (20 nM)
QNZ/ES (10 nM)
QNZ/ES (20 nM)
F
MDA-MB-231
Relative protein expression
DLAT oligomer
FDX1
HSP70
Vehicle
QNZ
ES
QNZ/ES
G
BT-549
Relative protein expression
DLAT oligomer
FDX1
HSP70
Vehicle
QNZ
ES
QNZ/ES
H
MDA-MB-231 xenograft
Tumor volume (mm³)
Days
Vehicle
QNZ
ES
QNZ/ES
I
MDA-MB-231 xenografted tumor
Vehicle
QNZ
ES
QNZ/ES
L
Ki-67/DAPI
Mito/DLAT/DAPI
FDX1/DAPI
p-IkBα/DAPI
Vehicle
QNZ
ES
QNZ/ES
Tumors from MDA-MB-231 xenografted mice
J
MDA-MB-231 xenograft
Tumor weight (g)
Vehicle
QNZ
ES
QNZ/ES
K
MDA-MB-231 xenograft
Body weight (g)
Days
Vehicle
QNZ
ES
QNZ/ES
M
Relative fluorescent intensity
Ki-67
DLAT
FDX1
p-IkBα
Vehicle
QNZ
ES
QNZ/ES

Figure S14. NF-κB inhibitor synergizes with cuproptosis inducer for cancer therapy

(A) Quantitative analysis of protein expression from main figure 7B. The results were analyzed using student *t* test (mean+/-SD, n = 3) (*P < 0.05, **P < 0.01, ***P < 0.001, and ****P < 0.0001). **(B-E)** MDA-MB-231 and BT-549 cells were treated with QNZ (5 μM) and elesclomol (ES) (MDA-MB-231, 5 nM and 10 nM; BT-549, 10 nM and 20 nM) individually or in combination for 24 hours, respectively, and the resulting cells were fixed and labeled with EdU (B and D), relative EdU-labeled cell numbers were normalized and plotted (C and E). The results were analyzed using student *t* test (mean+/-SD, n = 3) (*P < 0.05, **P < 0.01, ***P < 0.001, ****P < 0.0001). Bar indicates 100 μm. **(F-G)** Quantitative analyses of protein expression for main figure 7G. The results were analyzed using student *t* test (mean+/-SD, n = 3) (*P < 0.05, **P < 0.01, ***P < 0.001, and ****P < 0.0001). **(H-M)** MDA-MB-231 cells were subjected to xenograft assay. The mice bearing MDA-MB-231 xenografts were treated with QNZ and ES individually or in combination. The tumor size (H) and body weight of mouse (K) were monitored (mean+/-SD, n = 7) (*P < 0.05, **P < 0.01, ***P < 0.001, and ****P < 0.0001, *ANOVA* test). The tumors were dissected and weighed (I-J) (mean+/-SD, n = 7) (student *t* test). The tumors were subjected to IF assay (L) with indicated antibodies. The mitochondria were labelled using anti-TOM20 antibody. Bar indicates 50 μm. The fluorescent intensity was measured and plotted (M) (mean+/-SD, n = 3). The results were analyzed using student *t* test (*P < 0.05, **P < 0.01, ***P < 0.001, ****P < 0.0001).
